# Potential links between COVID-19 and periodontitis: a bioinformatic analysis based on GEO datasets

**DOI:** 10.1186/s12903-022-02435-4

**Published:** 2022-11-21

**Authors:** Churen Zhang, Yuzhe Sun, Min Xu, Chang Shu, Zhaoguo Yue, Jianxia Hou, Dongchen Ou

**Affiliations:** 1 Department of Stomatology, The First Affiliated Hospital of Xiamen University, School of Medicine, Xiamen University, 55 Zhenhai Avenue, Siming District, Xiamen, 361003 China; 2 Department of Periodontology, Peking University School and Hospital of Stomatology and National Center of Stomatology and National Clinical Research Center for Oral Diseases and National Engineering Laboratory for Digital and Material Technology of Stomatology and Beijing Key Laboratory of Digital Stomatology and Research Center of Engineering and Technology for Computerized Dentistry Ministry of Health and NMPA Key Laboratory for Dental Materials, Beijing, China

**Keywords:** 2019 Coronavirus disease, Periodontitis, Gene expression omnibus, Bioinformatics

## Abstract

**Background:**

2019 Coronavirus disease (COVID-19) is an infectious disease caused by the severe acute respiratory syndrome coronavirus 2 (SARS-CoV-2). The COVID-19 pandemic has already had a serious influence on human existence, causing a huge public health concern for countries all around the world. Because SARS-CoV-2 infection can be spread by contact with the oral cavity, the link between oral illness and COVID-19 is gaining traction. Through bioinformatics approaches, we explored the possible molecular mechanisms linking the COVID-19 and periodontitis to provide the basis and direction for future research.

**Methods:**

Transcriptomic data from blood samples of patients with COVID-19 and periodontitis was downloaded from the Gene Expression Omnibus database. The shared differentially expressed genes were identified. The analysis of Gene Ontology, Kyoto Encyclopedia of Genesand Genomes pathway, and protein–protein interaction network was conducted for the shared differentially expressed genes. Top 5 hub genes were selected through Maximal Clique Centrality algorithm. Then mRNA-miRNA network of the hub genes was established based on miRDB database, miRTarbase database and Targetscan database. The Least absolute shrinkage and selection operator regression analysis was used to discover possible biomarkers, which were then investigated in relation to immune-related genes.

**Results:**

Fifty-six shared genes were identified through differential expression analysis in COVID-19 and periodontitis. The function of these genes was enriched in regulation of hormone secretion, regulation of secretion by cell. Myozenin 2 was identified through Least absolute shrinkage and selection operator regression Analysis, which was down-regulated in both COVID-19 and periodontitis. There was a positive correlation between Myozenin 2 and the biomarker of activated B cell, memory B cell, effector memory CD4 T cell, Type 17 helper cell, T follicular helper cell and Type 2 helper cell.

**Conclusion:**

By bioinformatics analysis, Myozenin 2 is predicted to correlate to the pathogenesis and immune infiltrating of COVID-19 and periodontitis. However, more clinical and experimental researches are needed to validate the function of Myozenin 2.

**Supplementary Information:**

The online version contains supplementary material available at 10.1186/s12903-022-02435-4.

## Background

2019 Coronavirus disease (COVID-19) is an infectious disease caused by the severe acute respiratory syndrome coronavirus 2 (SARS-CoV-2) also that World Health Organization proclaimed a worldwide pandemic on March 11, 2020 [1]. The COVID-19 pandemic has already had a significant impact on human existence, because to its rapid spread and significant public health threat to nations all over the world [2]. Direct contact, droplet inhalation, or contact with oral, nasal, or ocular mucosa are the main ways that SARS-CoV-2 infection spreads. Patients with COVID-19 frequently report experiencing fever, coughing, dyspnea, muscle pains, and sleepiness [3]. Aside from efforts to reduce its economic burden, manage virus spread, and develop efficient preventative and treatment measures, there is a need to comprehend the disease's epidemiology and pathophysiologic components, as well as the risk factors that could lead various populations to severe clinical manifestations and an increased probability of death. Diabetes, hypertension, aging, immunodeficiency, and cardiovascular disease are among some of the risk factors for COVID-19 disease [4]. Furthermore, poor oral health, tooth loss, and periodontitis are regarded as common risk factors for adverse COVID-19 outcomes [5]. Some researchers have also indicated that excellent dental hygiene could help reduce the incidence of viral acute respiratory illness [6, 7]. In COVID-19 patients, these researches suggested a possible connection between oral and systemic health. Individuals with COVID-19 frequently experience oral symptoms such altered taste and smell, mouth ulcers, gingival pain, and bleeding [8]. The angiotensin-converting enzyme 2 (ACE2) receptor, which is a target of SARS CoV-2, has already been proved to be expressed in gingival epithelial cells, tongue taste cells, and salivary glands [9]. As a result, the oral cavity, sensitive to SARS-CoV-2, is regarded to be a possible area for human-to-human viral transmissions. A theoretical connection between poor dental status and COVID-19 severity and outcome was proposed in an observational analysis based on clinical oral examination and X-ray examination. This was because patients with poor dental health, such as caries and alveolar bone loss, had a higher rate of hospitalization [5].

"Bing cong kou ru," an ancient and well-known Chinese saying, was usually interpreted as "a closed mouth catches no flies." Several earlier investigations have discovered a link between periodontitis and many systemic disorders such as hypertension, diabetes, and premature birth in neonates [10, 11]. Periodontitis was common in the United States, with around 50% of persons over 30 years old having periodontitis and 8% having severe periodontitis [12]. In China, the prevalence of periodontitis was significantly greater. More than 90% of persons over the age of 35 had periodontitis at different levels, according to the Fourth National Oral Epidemiological Survey [13]. Periodontitis is a chronic inflammatory disease that results in the soft and hard tissues surrounding the teeth deteriorating, placing a significant load on global health, causing tooth displacement and even tooth loss [14]. Patients with periodontitis have gingival inflammation and bacteremia, which stimulate the host's inflammatory response and result in the release of various pro-inflammatory cytokines into the bloodstream, affecting general health [15]. Microbial invasion and the human immune inflammatory response were significant elements in periodontitis development, and genetic and epigenetic variables may impact periodontitis and other systemic disorders by modifying the host immunological response generated by periodontal pathogens [16]. COVID-19 has been linked to severe periodontitis. Gupta et al.[17] discovered that as the severity of periodontitis increased, so did hospital admissions, supplemental breathing requirements, and COVID-19 pneumonia in a cross-sectional research. Because SARS-CoV-2 infection can be spread by contact with the oral cavity, the link between oral illness and COVID-19 is gaining traction.

In recent years, bioinformatics and sequencing technology have advanced dramatically, and they are now commonly used to identify disease biomarkers [18]. Differently expressed mRNA, miRNA, and lncRNA can be found using sequencing or microarray technologies in a variety of disease samples, and bioinformatics can be utilized to further explore the function of the differentially expressed molecules in disease development. The Gene Expression Omnibus (GEO) database was used to download transcriptome information from blood samples of COVID-19 and periodontitis patients for the current investigation. Further through bioinformatics approaches, we explored the possible molecular mechanisms linking the two diseases to provide the basis and direction for future research.

## Materials & methods

### Data sources

The transcriptomes datasets of blood samples from patients with COVID-19 (GSE164805) and periodontitis (GSE12484) were obtained from the GEO database in NCBI (https://www.ncbi.nlm.nih.gov/geo/). GSE164805 was mRNA expression profile in GPL26963 (Agilent-085982 Arraystar human lncRNA V5 microarray), including 5 blood samples from healthy participants and 10 blood samples from COVID-19 patients. The peripheral blood mononuclear cells separated from blood were used for microarray analysis in GSE164805. GSE12484 was an expression profiling by array in GPL96 ([HG-U133A] Affymetrix Human Genome U133A Array), including 2 blood samples from chronic periodontitis patients and 2 blood samples from age/sex matched healthy controls. The peripheral blood neutrophils from blood were used for microarray analysis in GSE12484.

### Differentially expressed genes analysis

To find differentially expressed genes (DEGs), GEO2R, the GEO Online Analysis Tool (http://www.ncbi.nlm.nih.gov/geo/geo2r/), was used to identify DEGs in GSE164805 and GSE12484 between the diseased groups and control groups. DEGs were defined by the evaluation criteria of adjust *P*-value < 0.05 and |log2(FC)|> 2.0 for GSE164805. The threshold of *P*-value < 0.05 and |log2(FC)|> 2 was set for GSE12484. Then venn diagram was established to identify the intersected parts of the DEGs. Heatmap was used to show the 20 genes with the highest up- or down-regulation of expression each in COVID-19 and periodontitis, separately. BioGPS database (http://biogps.org/) was screened to find the tissue-specific expression of the intersected DEGs.

### Functional enrichment analysis of DEGs

To identify the biological function of intersected DEGs, Gene Ontology (GO) analysis and Kyoto Encyclopedia of Genesand Genomes (KEGG) pathways enrichment were conducted through Metascape database (https://metascape.org/). [19] GO analysis shows a description of genes in different dimensions and at different levels, which generally contains cellular component, biological process and molecular function. KEGG pathways enrichment systematically analyzes biological pathways that the genes participate in [20]. STRING database (https://string-db.org/) was applied to identify protein–protein interactions (PPI). Cytoscape was used to visualize the PPI. Furthermore, CytoHubba, a plug-in of Cytoscape, was applied to find the top 5 hub genes through Maximal Clique Centrality algorithm.

### Interacted mRNA-miRNA network

The miRDB database (https://mirdb.org/), [21] miRTarbase database (https://mirtarbase.cuhk.edu.cn/) [22] and Targetscan database (https://www.targetscan.org/) [23] were screened to identify the miRNAs interacted with the top 5 hub genes. The miRNA-mRNA network was built through the Cytoscape software.

### Interacted genes identified by LASSO regression analysis

To further identify the most valuable genes, the expression values of intersected DEGs in COVID-19 and periodontitis were used as characteristic values, which was used to conduct the “glmnet” package of R project (version 4.1.3). (Additional file: 1) Least absolute shrinkage and selection operator (LASSO) Regression Analysis were applied, and then the DEGs identified by calculation were the potential biomarkers. After that, the expression values of the potential biomarkers in all COVID-19 and periodontitis samples were used for the Wilcoxon test to show the expression level between disease samples and control samples.

### The correlation between the identified potential biomarkers and immune cells

The immune related genes of different immune cells were obtained from the previously published literature [24]. The 782 immune-related genes that belonged to biomarkers of adaptive and innate immune cells were downloaded. (Additional file: 2) The expression value of the 782 immune related genes in COVID-19 and periodontitis were extracted. The Pearson correlation test was used to determine the expression association between the putative biomarkers discovered by LASSO regression and the 782 immune relevant genes.

## Results

### DEGs in COVID-19 and periodontitis

**Fig. 1:**
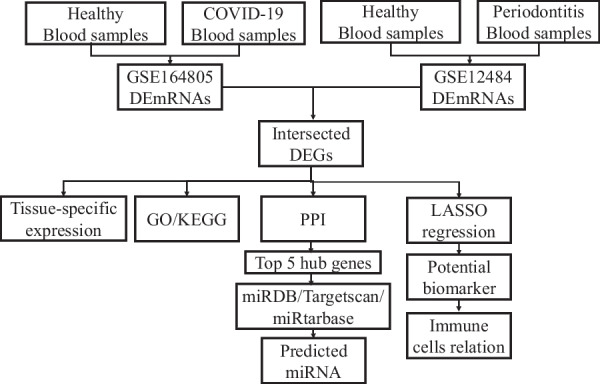
Research flow. The process of the present study

**Fig. 2:**
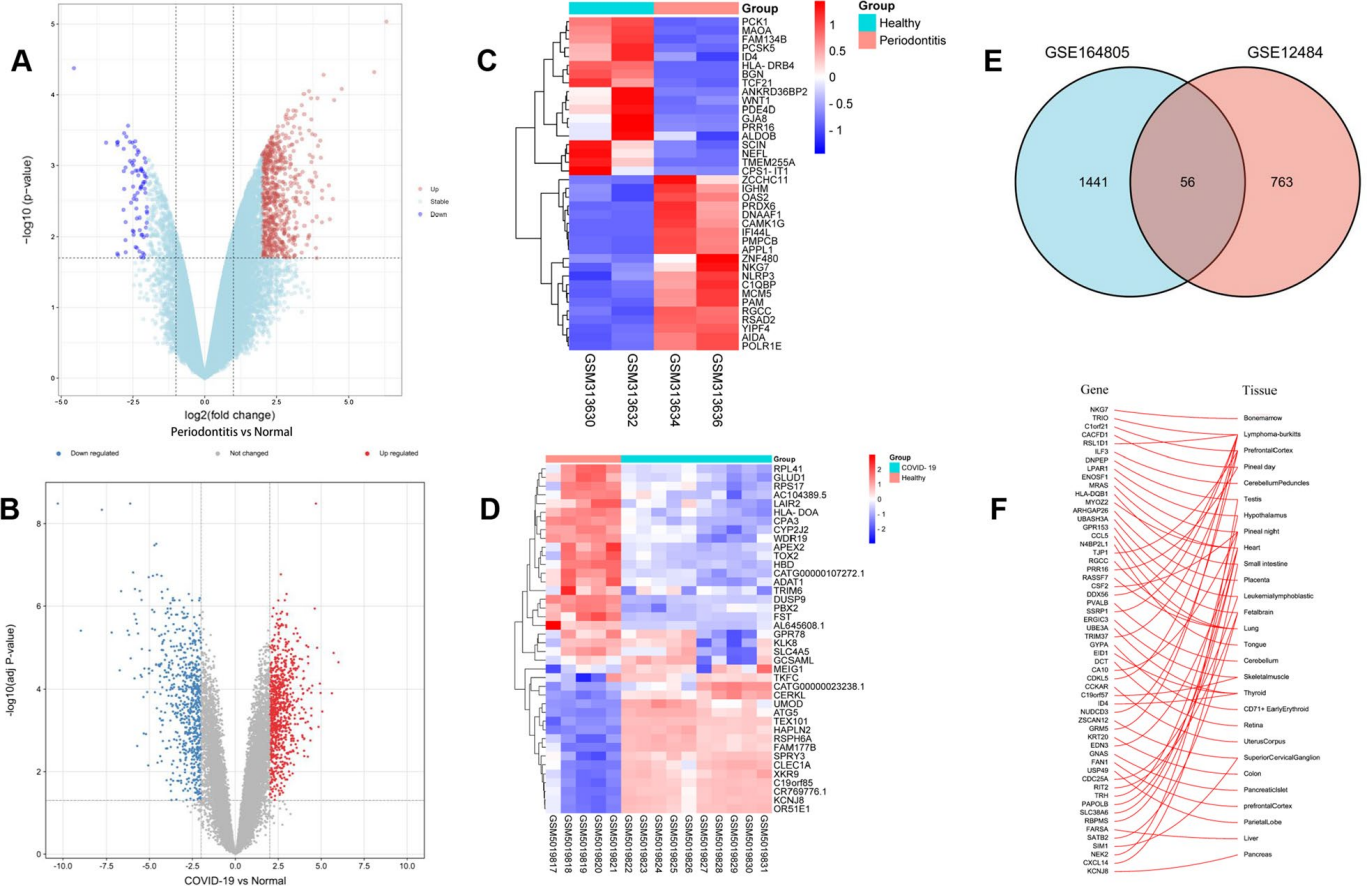
DEGs analysis in COVID-19 and periodontitis. **A** Volcano plot of mRNAs expression level in GSE12484. Threshold of *P*-value < 0.05 and |log2(FC)|> 2 was set. The red dots represented significantly highly expressed genes, and the blue dots represented significantly lowly expressed genes; **B** Volcano plot of mRNAs expression level in GSE164805. The evaluation criteria included adjust *P*-value < 0.05 and |log2(FC)|> 2.0. The red dots represented significantly highly expressed genes, and the blue dots represented significantly lowly expressed genes; **C** The heatmap showed the expression of top 20 up-regulated mRNAs and down-regulated mRNAs in GSE12484; **D** The heatmap showed the expression of top 20 up-regulated mRNAs and down-regulated mRNAs in GSE164805; **E** Then venn diagram showed the overlapping parts of GSE164805 and GSE12484: **F** The tissue-specific expression of the intersected DEGs

The flowchart of this research is shown in Fig. 1. In GSE12484, the 819 differentially expressed genes (730 up-regulated genes and 89 down-regulated genes) were obtained, and the 1526 differentially expressed genes (855 up-regulated genes and 671 down-regulated genes) are acquired in GSE164805. The expression profiles of all genes of GSE12484 and GSE164805 are displayed in Fig. 2A, 2B. The expression level of 40 genes with the highest |log2(FC)|-values in GSE12484 and GSE164805 are displayed in Fig. 2C, 2D. The 56 genes are found differentially expressed in both COVID-19 and periodontitis. (Fig. 2E) Tissues with the highest intersected DEGs expression are shown in Fig. 2F.

### Functional enrichment analysis

**Fig. 3:**
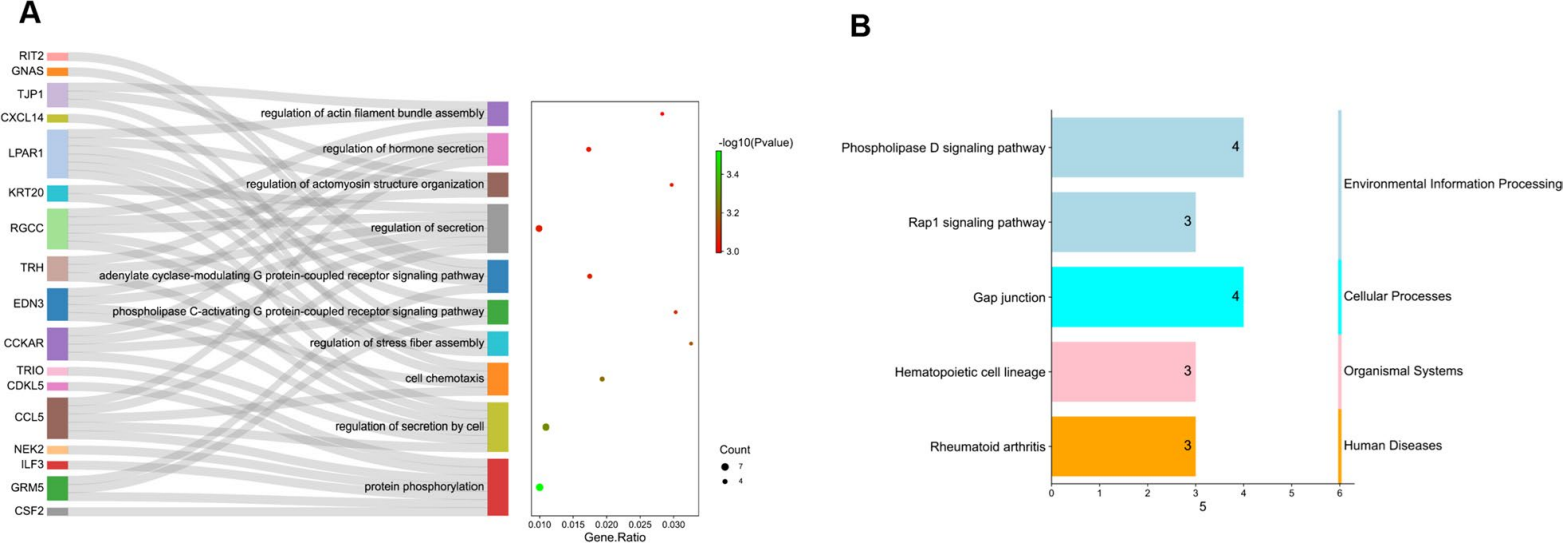
GO/KEGG analysis of the intersected DEGs between COVID-19 and periodontitis. **A** The top 10 terms of GO analysis of intersected DEGs; **B** The KEGG pathway analysis of intersected DEGs

The function of intersected DEGs is enriched in regulation of actin filament bundle assembly, regulation of hormone secretion, regulation of secretion by cell, protein phosphorylation, cell chemotaxis. (Fig. 3A) Through KEGG analysis, the intersected DEGs are found to be involved in Gap junction, Phospholipase D signaling pathway, Rap1 signaling pathway, Rheumatoid arthritis. (Fig. 3B).

### Construction of PPI network and miRNA network of intersected DEGs

**Fig. 4:**
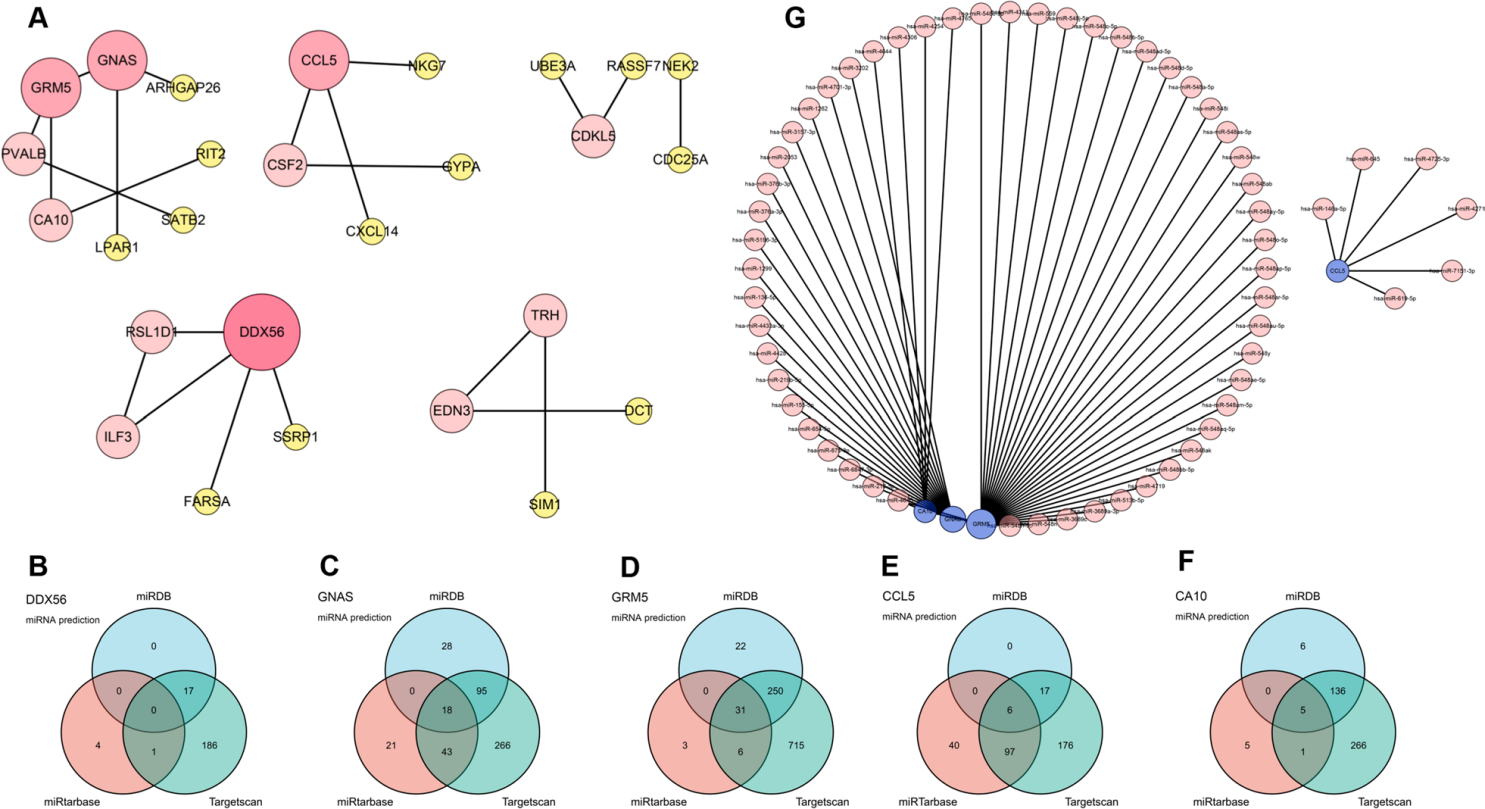
PPI network and mRNA-miRNA network of the intersected DEGs between COVID-19 and periodontitis. **A** PPI network of intersected DEGs, with DDX56, GNAS, GRM5, CCL5 and CA10 as the top 5 hub genes; (**B–F**) The venn diagram showed the overlapping parts of miRNA interacting with DDX56, GNAS, GRM5, CCL5 and CA10 from miRDB database, miRTarbase database and Targetscan database; **G** The mRNA-miRNA network of GNAS, GRM5, CCL5 and CA10. There was no intersected miRNA interacting with DDX56 from miRDB database, miRTarbase database and Targetscan database. DDX56 was not included in the mRNA-miRNA network

The PPI network of the intersected DEGs is shown in Fig. 4A. The intersected DEGs with no interactions to other proteins were not displayed. The top 5 hub genes are DEAD-Box Helicase 56 (DDX56), GNAS Complex Locus (GNAS), Glutamate activates metabotropic receptor 5 (GRM5), C–C Motif Chemokine Ligand 5 (CCL5) and Carbonic Anhydrase 10 (CA10). GRM5 interacts with GNAS and CA10. The miRNAs, that are predicted to interact with each hub gene in the miRDB database, miRTarbase database and Targetscan database, are taken the intersection. (Fig. 4B–F) After that, 0 miRNA interacted with DDX56 is left. The 18 miRNAs are finally identified for GNAS. The number of miRNA interacted with GRM5, CCL5 and CA10 are 31, 6 and 5, separately. Then, the miRNA network of intersected DEGs is constructed. (Fig. 4G) The hsa-miR-4645-3p is found to interact with GRM5 and CA10.

### LASSO regression analysis of the intersected DEGs

**Fig. 5:**
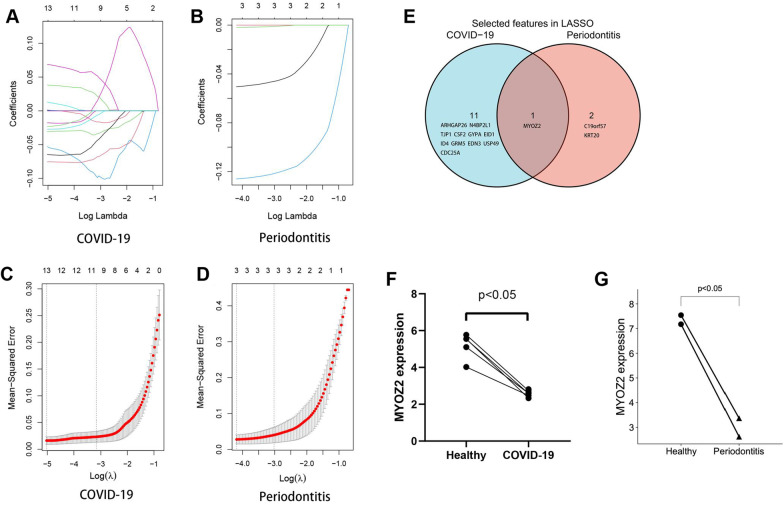
LASSO regression analysis of the intersected DEGs. **A, B** Change curves of characteristic gene for COVID-19 and periodontitis. Logarithm of the lambdas and variable coefficient were the horizontal and vertical coordinates, separately. The values on the top of the coordinate axis represented the number of variable genes with variable coefficient was not 0 under the log value of the current lambda, which might be more valuable in the gene dataset. **C, D** The outcomes of cross-referencing the lambda result. In the illustration, there are two dashed lines: lambda.min with the smallest mean square error and lambda. 1se with the standard deviation from the mean square error; **E** The venn diagram showed the overlapping parts of the results of LASSO analysis of COVID-19 and periodontitis; **F, G** The expression of MYOZ3 in COVID-19 and periodontitis samples were tested by the Wilcoxon test

There are 56 intersected DEGs, and LASSO regression analysis is applied to further explore the potential biomarkers of both COVID-19 and periodontitis. (Fig. 5A–D) Through screening, 11 intersected DEGs are identified in COVID-19, and 3 intersected DEGs are identified in periodontitis. They share Myozenin 2 (MYOZ2) (Fig. 5E). The expression values of MYOZ2 in COVID-19 and periodontitis are shown in scatter plot, and the expression of MYOZ2 in COVID-19 and periodontitis is lower than that in the normal samples. (Fig. 5F, G).

### Correlation between MYOZ2 and immune cells

**Fig. 6:**
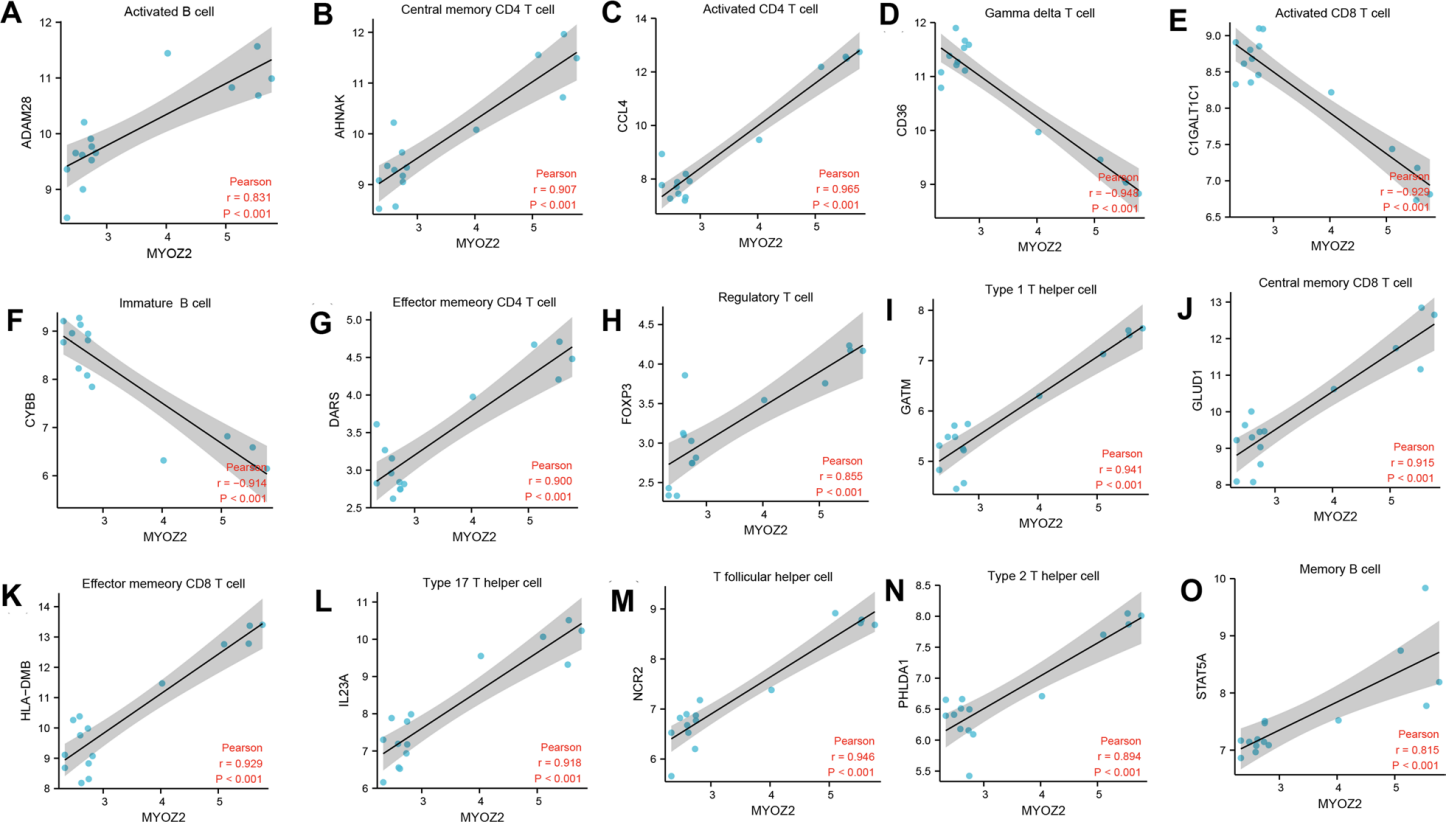
The correlation between MYOZ3 and immune related genes in COVID-19. (**A–O**) the correlation of gene expression between MYOZ3 and the biomarker of activated B cell, central memory CD4 T cell, activated CD4 T cell, gamma delta T cell, activated CD8 T cell, immature B cell, effector memory CD4 T cell, regulatory T cell, Type 1 T helper cell, central memory CD8 T cell, effector memory CD8 T cell, Type 17 T helper cell, T follicular helper cell, Type 2 T helper cell, memory B cell

**Fig. 7:**
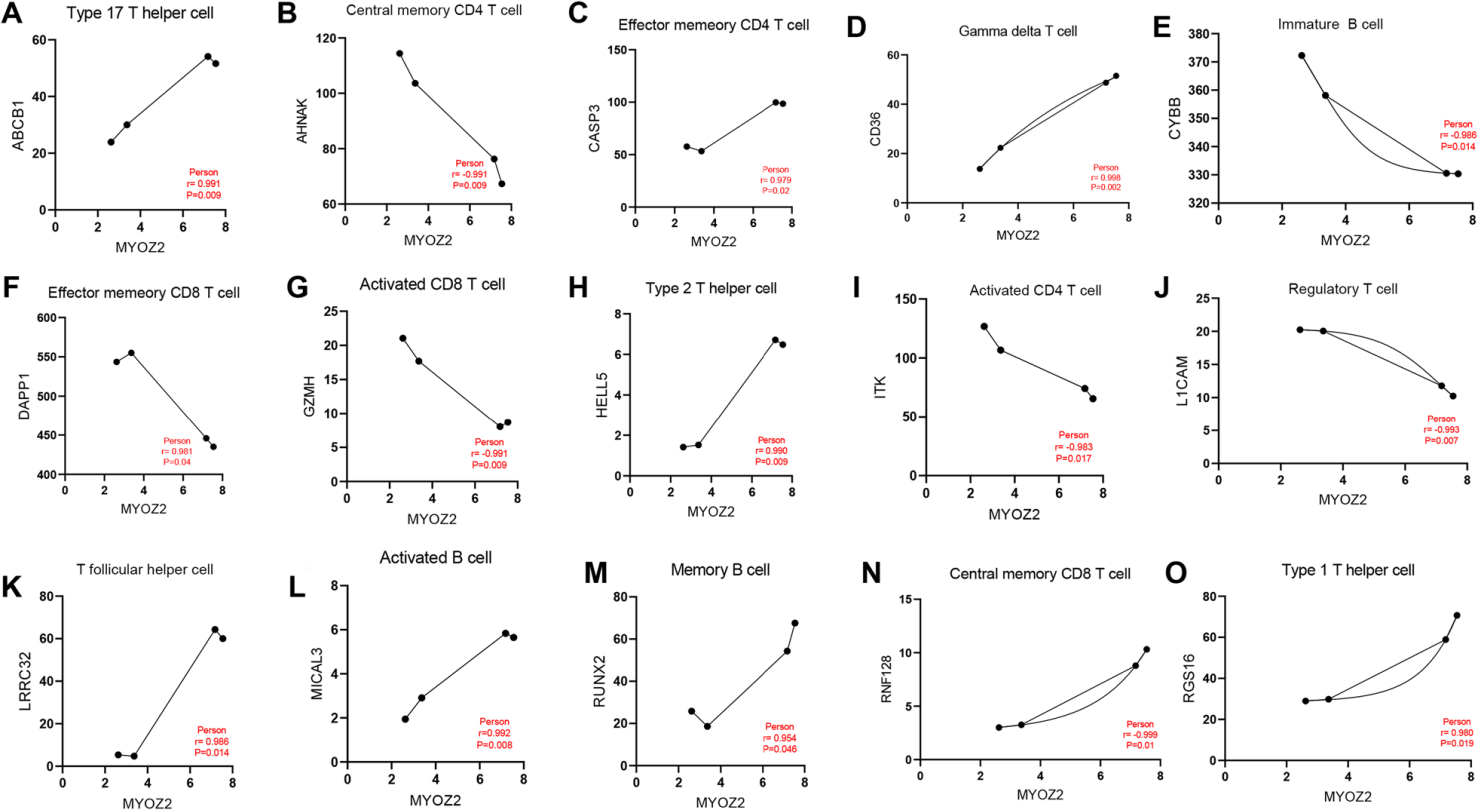
The correlation between MYOZ3 and immune related genes in periodontitis. (**A–O**) the correlation of gene expression between MYOZ3 and the biomarker of Type 17 T helper cell, central memory CD4 T cell, effector memory CD4 T cell, gamma delta T cell, immature B cell, effector memory CD8 T cell, activated CD8 T cell, Type 2 T helper cell, activated CD4 T cell, regulatory T cell, T follicular helper cell, activated B cell, memory B cell, central memory CD8 T cell, Type 1 T helper cell

In the gene profiles of COVID-19, MYOZ2 is significantly positively correlated to ADAM Metallopeptidase Domain 28 (ADAM28), the biomarker of Activated B cell, C–C Motif Chemokine Ligand 4 (CCL4), the biomarker of Activated CD4 T cell, C1GALT1 Specific Chaperone 1 (C1GALT1C1), the biomarker of Activated CD8 T cell, and many other biomarkers of adaptive immune cells and innate immune cells. (Fig. 6, Additional file: 3) In the gene profiles of periodontitis, MYOZ2 is significantly positively correlated to ATP Binding Cassette Subfamily B Member 1 (ABCB1), the biomarker of Type 17 T help cells, Caspase 3 (CASP3), the biomarker of effector memory CD4 T cell, CD36 Molecule (CD36), the biomarker of Gamma delta T cell, and many other biomarkers of adaptive immune cells and innate immune cells. (**Fig. ****7****,** Additional file: 4) In addition, bioinformatic procedure of this study was listed step-by-step in Additional file: 5

## Discussion

By boosting the release of pro-inflammatory cytokines and encouraging neutrophils to enter the periodontal lesion, microbial imbalance in periodontitis triggers the host immune response against pathogenic bacteria [25]. Periodontal pathogenic bacteria may enter the lungs via the oral-pulmonary respiratory axis or salivary transmission, and they exacerbate the severe pulmonary infection induced by SARS-CoV-2 by stimulating the release of inflammatory cytokines [26]. Additionally, periodontal inflammation promotes the inflammatory and innate immune responses by facilitating the production of reactive oxygen species, which damages DNA in cells [27]. Moreover, induced DNA damage can cause lung epithelial cell malfunction, which favors SARS-CoV-2 invasion [28]. Even though several pertinent studies on the connection between COVID-19 and periodontitis have been done, more research are needed to be done on the probable mechanisms and biomarkers that connect the two diseases.

The present study find that 56 genes are significantly co-expressed in COVID-19 and periodontitis. The function of these genes is enriched in regulation of hormone secretion, regulation of secretion by cell, protein phosphorylation, cell chemotaxis. GO analysis shows that cellular ion homeostasis is mediated by Lysophosphatidic acid receptor 1 (LPAR1), Endothelin 3 (EDN3), GRM5, CCL5, and Ubiquitin Protein Ligase E3A (UBE3A). Serum levels of hepcidin, a systemic iron-regulatory hormone, were greater in severe COVID-19 cases [29]. Hepcidin is upregulated by IL-6 and may have a role in systemic inflammation caused by periodontitis [30]. KEGG analysis indicates that DEGs are found to be enriched in Gap junction. Infection with SARS-CoV-2 increases soluble E-cad protein and causes dysregulation of other cell adhesion proteins, affecting tight-, adherens-, and gap-junctions in pulmonary tissue [31]. A junctional epithelium connects the teeth and gingiva. In periodontitis, microbial stimulation affects the usual defensive mechanisms in periodontal tissue, causing disruption of the junctional epithelium and promoting the entry of periodontal pathogens and their metabolites into the periodontal tissue [32].

The top 5 hub genes are identified through Maximal Clique Centrality algorithm, including DDX56, GNAS, GRM5, CCL5 and CA10. DDX56 was involved in a variety of cellular activities involving RNA secondary structure modification, including translation initiation, nuclear splicing, and ribosome and spliceosome assembly [33]. GNAS may suppress the adenylyl cyclase-stimulating activity of G(s) subunit alpha, which is generated from the same locus but in a different open reading frame [34]. An epigenome-wide DNA methylation analysis by Zhou et al. revealed that GNAS may play an important role in the course of COVID-19 [35]. GRM5 causes a conformational shift that initiates signaling via guanine nucleotide-binding proteins (G proteins) and affects the activity of downstream effectors [36]. One of the key HIV-suppressive factors produced by CD8 + T-cells, CCL5 is engaged in immunoregulatory and inflammatory processes [37]. Lipopolysaccharide induced the expression of CCL5 in CD14 + sorted cells from gingival crevicular fluid of periodontitis patients [38]. SARS-CoV-2 infection induced CCL2, CCL5 and IL-6 release from placental cells, which might promote the cytokine storm in pregnant women with COVID-19 [39]. CA10 is regarded as a functional gene in the central nervous system, and Chondroblastoma and Non-Suppurative Otitis Media are two diseases linked to CA10 [40].

Myozenin 2 (MYOZ2) is identified by LASSO regression analysis. MYOZ2 may function as intracellular binding proteins that connect Z line proteins, and be essential for the control of calcineurin signaling [41]. It is found in our study that MYOZ2 is down-regulated in both periodontitis and COVID-19. Further we explore the correlation between MYOZ2 and immune-related genes. The results shows that there is a positive correlation between MYOZ2 and the biomarker of activated B cell, memory B cell, effector memory CD4 T cell, Type 17 helper cell (Th17), T follicular helper cell and Type 2 helper cell (Th2). The down-regulation of MYOZ2 might lead to the less infiltration of the mentioned immune cells in periodontitis and COVID-19 to further affect the immune response during the pathogenesis of the two diseases. B cells' primary job is to create antibodies that mediate humoral immune responses, and activated B cells can deliver soluble antigens [42]. Antibodies produced by B cells inhibit pathogens from attaching to target cells, stopping further infection. Moreover, after the antibody binds to the pathogen's surface, the complement is activated, and an antigen–antibody-complement complex is formed, bringing the pathogen to the phagocyte and making it easier to devour [43]. Various immune cell infiltrations were present in the periodontitis lesions, with B cells accounting for approximately 18% of all infiltrating leukocytes [16]. B cell insufficiency causes more severe alveolar bone deterioration in mice with experimental periodontitis, which is related with increased osteoclast activity [44]. Using single-cell sequencing methods, Zhang et al.[45] discovered that the fraction of memory B cell subsets in COVID-19 patients was significantly lower than in healthy people. Furthermore, a single B cell cloning technique can be used to select monoclonal antibodies against SARS-CoV-2, which has implications for the diagnosis and treatment of COVID-19 disease. [43] T cells are important in the immune response to periodontitis and COVID-19. In response to the activation of a periodontal pathogen, periodontal tissue expresses chemokines, and chemotactic polymorphonuclear granulocytes and monocytes migrate to the site of inflammation [46]. When inflammation persists, bacterial products stimulate antigen-presenting cells (APCs) to contact undifferentiated T cells, activate T cells, and promote the differentiation of various T cell subsets [47]. Activation of Th2 can lead to activation of B cells, further increasing antibody production, and activation of Th17 [47]. Dendritic cells (DCs) and macrophages can engulf virus-infected cells during the early stages of SARS-CoV-2 infection, triggering T-cell responses through antigen presentation. CD4 + T cells then drive B cells to make virus-specific antibodies, while cytotoxic CD8 + T cells attack virus-infected cells [48]. In addition, it was reported that more than 70% of COVID-19 convalescent patients had SARS-CoV-2-specific T cells [49].

However, there are no research about the function of MYOZ2 in periodontitis and COVID-19. Our present study provides a new perspective for the potential links between periodontitis and COVID-19. More relevant studies are needed.

## Conclusion

By bioinformatics analysis, MYOZ2 is predicted to correlate to the pathogenesis and immune infiltrating of COVID-19 and periodontitis. However, more clinical and experimental researches are needed to validate the function of MYOZ2.

## Supplementary Information

**Additional file 1:** Code of lasso.

**Additional file 2:** Immune cell biomarker.

**Additional file 3:** COVID19 immune.

**Additional file 4:** Periodontitis immune.

**Additional file 5:** Bioinformatic procedure.

## Data Availability

The transcriptomes datasets of blood samples from patients with COVID-19 (GSE164805) and periodontitis (GSE12484) were obtained from the GEO database in NCBI (https://www.ncbi.nlm.nih.gov/geo/).

## Abbreviations

COVID-19: 2019 Coronavirus disease
SARS-CoV-2: Severe acute respiratory syndrome coronavirus 2
GEO: Gene expression omnibus
DEGs: Differentially expressed genes
GO: Gene ontology
KEGG: Kyoto encyclopedia of genesand genomes
PPI: Protein–protein interactions
LASSO: Least absolute shrinkage and selection operator
DDX56: DEAD-box helicase 56
GNAS: GNAS complex locus
GRM5: Glutamate activates metabotropic receptor 5
CCL5: C–C motif chemokine ligand 5
CA10: Carbonic anhydrase 10
MYOZ2: Myozenin 2
ADAM28: ADAM metallopeptidase domain 28
CCL4: C–C motif chemokine ligand 4
C1GALT1C1: C1GALT1 Specific chaperone 1
ABCB1: ATP binding cassette subfamily B member 1
CASP3: Caspase 3
CD36: CD36 molecule
