# Supplementary material for: Potential links between COVID-19 and periodontitis: a bioinformatic analysis based on GEO datasets
Source: BMC Oral Health. 2022 Nov 21;22:520. doi: 10.1186/s12903-022-02435-4 (PMC9682728; doi:10.1186/s12903-022-02435-4)
Supplement: Supplementary file 1 — Additional file 1: Code of lasso. [file 12903_2022_2435_MOESM1_ESM.docx]

**Supplement 1**

**The codes used for LASSO regression analysis**

library(glmnet)

library(foreign)

library(ggplot2)

setwd("D:\\111\\read")

name <- c("Type", "NKG7", "TRIO", "C1orf21", "CACFD1", "RSL1D1", "ILF3", "DNPEP", "LPAR1", "ENOSF1", "MRAS", "HLA-DQB1", "MYOZ2", "ARHGAP26", "UBASH3A", "GPR153", "CCL5", "N4BP2L1", "TJP1", "RGCC", "PRR16", "RASSF7", "CSF2", "DDX56", "PVALB", "SSRP1", "ERGIC3", "UBE3A", "TRIM37", "GYPA", "EID1", "DCT", "CA10", "CDKL5", "CCKAR", "C19orf57", "ID4", "NUDCD3", "ZSCAN12", "GRM5", "KRT20", "EDN3", "GNAS", "FAN1", "USP49", "CDC25A", "RIT2", "TRH", "PAPOLB", "SLC38A6", "RBPMS", "FARSA", "SATB2", "SIM1", "NEK2", "CXCL14", "KCNJ8")

bc <- read.table("covid.txt",header = ,sep = "\t",col.names = name,skip = 1)

bc

y <- as.matrix(bc[,1])

x <- as.matrix(bc[,c(2:57)])

fi = glmnet(x,y,family = "gaussian",alpha = 1,nlambda = 100)

fi

plot(fi,xvar="lambda",label = TRUE)

library(Matrix)

cv.fit <- cv.glmnet(x,y,family = "gaussian")

cv.fit

plot(cv.fit)

a <- coef(cv.fit$fit, s = "lambda.min")

a

predict(fi,newx = x[2:5,],type = "response")

cvfit=cv.glmnet(x,y)

plot(cvfit)

cvfit$lambda.min

cvfit$lambda.1se

l.corf2 <- coef(cvfit$glmnet.fit,s=0.006559348,exact = F)

l.corf1 <- coef(cvfit$glmnet.fit,s=0.04216397,exact = F)

l.corf1

l.corf2

mod <- glm(Type~CACFD1 + DNPEP + MYOZ2 + ARHGAP26 + N4BP2L1 + TJP1 + CSF2 + GRM5 + USP49 + FARSA,family = "binomial",data = bc)

summary(mod)

mod <- glm(Type~DNPEP + MYOZ2 + ARHGAP26 + N4BP2L1 + TJP1 + CSF2 + GYPA + EID1 + ID4 + GRM5 + EDN3 + USP49 + CDC25A,family = "binomial",data = bc)

summary(mod)

bc <- read.table("periodontitis.txt",header = ,sep = "\t",col.names = name,skip = 1)

bc

y <- as.matrix(bc[,1])

x <- as.matrix(bc[,c(2:57)])

fi = glmnet(x,y,family = "gaussian",alpha = 1,nlambda = 100)

fi

plot(fi,xvar="lambda",label = TRUE)

cv.fit <- cv.glmnet(x,y,family = "gaussian")

cv.fit

plot(cv.fit)

a <- coef(cv.fit$fit, s = "lambda.min")

a

predict(fi,newx = x[2:5,],type = "response")

cvfit=cv.glmnet(x,y)

plot(cvfit)

cvfit$lambda.min

cvfit$lambda.1se

l.corf2 <- coef(cvfit$glmnet.fit,s=0.006559348,exact = F)

l.corf1 <- coef(cvfit$glmnet.fit,s=0.04216397,exact = F)

l.corf1

l.corf2
