# Supplementary material for: Potential links between COVID-19 and periodontitis: a bioinformatic analysis based on GEO datasets
Source: BMC Oral Health. 2022 Nov 21;22:520. doi: 10.1186/s12903-022-02435-4 (PMC9682728; doi:10.1186/s12903-022-02435-4)
Supplement: Supplementary file 3 — Additional file 3: COVID19 immune. [file 12903_2022_2435_MOESM3_ESM.docx]

**Supplement 3**

**The immune related genes that were significantly associated with MYOZ3 through Pearson test in COVID-19.**

| Immune type | Immune cells | Pearson Correlation | MYOZ2 | |
| --- | --- | --- | --- | --- |
|  |  |  | r | P |
| Adaptive | Activated B cell | ADAM28 | 0.831 | 0.001 |
|  | Activated CD4 T cell | CCL4 | 0.904 | 0.0001 |
|  |  | DUSP2 | 0.809 | 0.0003 |
|  |  | ETS1 | 0.897 | 0.0001 |
|  |  | KNTC1 | -0.841 | 0.0001 |
|  | Activated CD8 T cell | C1GALT1C1 | -0.929 | 0.0001 |
|  |  | CD37 | 0.838 | 0.0001 |
|  |  | CD3D | 0.810 | 0.0003 |
|  |  | GNLY | 0.873 | 0.0001 |
|  |  | GZMK | 0.808 | 0.0003 |
|  |  | MPZL1 | -0.887 | 0.0001 |
|  |  | NKG7 | 0.824 | 0.0002 |
|  |  | PIK3IP1 | 0.841 | 0.0001 |
|  |  | TIMM13 | 0.909 | 0.0001 |
|  |  | ZAP70 | 0.881 | 0.0001 |
|  | Central memory CD4 T cell | AHNAK | 0.907 | 0.0001 |
|  |  | BZW2 | 0.807 | 0.0003 |
|  |  | CYLD | 0.873 | 0.0001 |
|  |  | GSS | -0.822 | 0.0002 |
|  |  | IFITM2 | 0.835 | 0.0001 |
|  |  | NDUFB9 | 0.902 | 0.0001 |
|  |  | XRCC6 | 0.822 | 0.0002 |
|  | Central memory CD8 T cell | FCGR3B | -0.848 | 0.0001 |
|  |  | GLUD1 | 0.915 | 0.0001 |
|  |  | NOL11 | 0.905 | 0.0001 |
|  |  | TOX4 | 0.878 | 0.0001 |
|  |  | UBA52 | -0.907 | 0.0001 |
|  | Effector memeory CD4 T cell | DARS | 0.900 | 0.0001 |
|  |  | NEFL | -0.855 | 0.0001 |
|  |  | PDGFRL | 0.844 | 0.0001 |
|  |  | SIGLEC14 | -0.833 | 0.0001 |
|  |  | TPK1 | 0.848 | 0.0001 |
|  | Effector memeory CD8 T cell | GZMM | 0.890 | 0.0001 |
|  |  | HLA-DMB | 0.929 | 0.0001 |
|  |  | SIK1 | 0.847 | 0.0001 |
|  | Gamma delta T cell | C1orf54 | -0.880 | 0.0001 |
|  |  | CD36 | 0.948 | 0.0001 |
|  |  | CCL13 | 0.830 | 0.0001 |
|  | Immature B cell | CYBB | -0.914 | 0.0001 |
|  | Memory B cell | STAT5A | 0.815 | 0.0002 |
|  | Regulatory T cell | CCL3L1 | 0.838 | 0.0001 |
|  |  | FOXP3 | 0.855 | 0.0001 |
|  |  | MS4A6A | 0.814 | 0.0002 |
|  | T follicular helper cell | NCR2 | 0.946 | 0.0001 |
|  |  | NRP1 | -0.914 | 0.0001 |
|  |  | PDCD6 | 0.816 | 0.0002 |
|  |  | PRDX1 | -0.904 | 0.0001 |
|  | Type 1 T helper cell | CD48 | 0.865 | 0.0001 |
|  |  | CD6 | 0.886 | 0.0001 |
|  |  | CD7 | -0.817 | 0.0002 |
|  |  | CD96 | 0.854 | 0.0001 |
|  |  | GATM | 0.941 | 0.0001 |
|  |  | SH3KBP1 | 0.904 | 0.0001 |
|  |  | SIT1 | 0.852 | 0.0001 |
|  |  | SKAP1 | 0.844 | 0.0001 |
|  |  | METRNL | 0.810 | 0.0002 |
|  |  | DUSP14 | -0.931 | 0.0001 |
|  | Type 17 T helper cell | IL23A | 0.917 | 0.0001 |
|  |  | ILDR1 | -0.890 | 0.0001 |
|  |  | CD40 | -0.853 | 0.0001 |
|  | Type 2 T helper cell | PHLDA1 | 0.894 | 0.0001 |
|  |  | LAIR2 | 0.850 | 0.0001 |
| Innate | Activated dendritic cell | NOS2 | 0.976 | 0.0001 |
|  |  | RHOA | 0.868 | 0.0001 |
|  |  | SLC25A37 | 0.862 | 0.0001 |
|  |  | SIGLEC5 | -0.835 | 0.0001 |
|  | CD56bright natural killer cell | ABAT | -0.877 | 0.0001 |
|  |  | MLST8 | 0.923 | 0.0001 |
|  |  | CREB3L4 | 0.950 | 0.0001 |
|  |  | CSTA | 0.809 | 0.0003 |
|  |  | CSTB | 0.895 | 0.0001 |
|  |  | FST | 0.934 | 0.0001 |
|  |  | GATA2 | 0.821 | 0.0002 |
|  |  | CYP27B1 | 0.848 | 0.0001 |
|  | CD56dim natural killer cell | HLA-E | 0.952 | 0.0001 |
|  | Eosinophil | RRP12 | 0.902 | 0.0001 |
|  |  | GPR183 | 0.882 | 0.0001 |
|  |  | NR4A3 | 0.881 | 0.0001 |
|  | Immature dendritic cell | AMT | 0.903 | 0.0001 |
|  |  | RRAGD | 0.823 | 0.0002 |
|  | Macrophage | CCL14 | 0.836 | 0.0001 |
|  |  | CCL26 | 0.831 | 0.0001 |
|  |  | IGSF6 | 0.807 | 0.0003 |
|  |  | FES | 0.837 | 0.0001 |
|  | Mast cell | CPA3 | 0.889 | 0.0001 |
|  |  | CTSG | 0.903 | 0.0001 |
|  |  | ITGA9 | 0.854 | 0.0001 |
|  |  | SIGLEC8 | 0.872 | 0.0001 |
|  | MDSC | CXCR4 | 0.921 | 0.0001 |
|  |  | GPSM3 | 0.807 | 0.0003 |
|  |  | PSAP | 0.851 | 0.0001 |
|  | Monocyte | ATP6V1B2 | 0.856 | 0.0001 |
|  |  | CFL1 | -0.971 | 0.0001 |
|  |  | MARCKSL1 | 0.878 | 0.0001 |
|  |  | TMBIM6 | 0.819 | 0.0002 |
|  | Natural killer cell | CTSZ | 0.980 | 0.0001 |
|  |  | DAXX | 0.866 | 0.0001 |
|  |  | ERBB3 | 0.813 | 0.0002 |
|  |  | FAM49A | 0.909 | 0.0001 |
|  |  | FCGR1A | 0.801 | 0.0003 |
|  |  | FGF18 | 0.858 | 0.0001 |
|  |  | FUT5 | 0.907 | 0.0001 |
|  | Natural killer T cell | CNPY3 | 0.884 | 0.0001 |
|  |  | CNPY4 | 0.838 | 0.0001 |
|  |  | CSF2 | 0.841 | 0.0001 |
|  |  | IL32 | 0.819 | 0.0002 |
|  |  | NFATC2IP | 0.916 | 0.0001 |
|  |  | KIR3DL2 | 0.871 | 0.0001 |
|  |  | TREM2 | 0.861 | 0.0001 |
|  |  | CLEC1A | 0.946 | 0.0001 |
|  |  | THBD | 0.844 | 0.0001 |
|  |  | PDPN | 0.909 | 0.0001 |
|  | Neutrophil | STEAP4 | -0.919 | 0.0001 |
|  | Plasmacytoid dendritic cell | HIGD1A | 0.896 | 0.0001 |
|  |  | FCAR | 0.836 | 0.0001 |
|  |  | IGF1 | -0.940 | 0.0001 |
|  |  | EMP3 | 0.918 | 0.0001 |
