# Supplementary material for: Potential links between COVID-19 and periodontitis: a bioinformatic analysis based on GEO datasets
Source: BMC Oral Health. 2022 Nov 21;22:520. doi: 10.1186/s12903-022-02435-4 (PMC9682728; doi:10.1186/s12903-022-02435-4)
Supplement: Supplementary file 4 — Additional file 4: Periodontitis immune. [file 12903_2022_2435_MOESM4_ESM.docx]

**Supplement 4**

**The immune related genes that were significantly associated with MYOZ3 through Pearson test in periodontitis.**

| Immune type | Immune cells | Person correlation | MYOZ2 | |
| --- | --- | --- | --- | --- |
|  |  |  | r | P |
| Adaptive | Activated B cell | ADAM28 | 0.960 | 0.040 |
|  |  | CD19 | 0.987 | 0.013 |
|  |  | IGHM | -0.974 | 0.026 |
|  |  | MICAL3 | 0.992 | 0.008 |
|  |  | SPIB | 0.981 | 0.019 |
|  | Activated CD4 T cell | AIM2 | 0.952 | 0.048 |
|  |  | CCL5 | -0.954 | 0.047 |
|  |  | DUSP2 | -0.951 | 0.049 |
|  |  | ETS1 | -0.981 | 0.019 |
|  |  | ITK | -0.983 | 0.017 |
|  |  | KIF11 | 0.983 | 0.017 |
|  |  | SAMSN1 | -0.971 | 0.029 |
|  | Activated CD8 T cell | CCT6B | 0.959 | 0.041 |
|  |  | GZMH | -0.991 | 0.009 |
|  |  | MPZL1 | 0.960 | 0.040 |
|  | Central memory CD4 T cell | AHNAK | -0.991 | 0.009 |
|  |  | CD63 | 0.965 | 0.035 |
|  |  | CYLD | -0.990 | 0.010 |
|  |  | FYN | -0.987 | 0.013 |
|  |  | IFITM2 | 0.989 | 0.011 |
|  |  | ITGB1 | -0.965 | 0.035 |
|  | Central memory CD8 T cell | RNF128 | 0.990 | 0.010 |
|  | Effector memeory CD4 T cell | CASP3 | 0.979 | 0.021 |
|  |  | EZH2 | -0.961 | 0.039 |
|  | Effector memeory CD8 T cell | CCR5 | 0.953 | 0.048 |
|  |  | CD55 | -0.977 | 0.023 |
|  |  | DAPP1 | -0.981 | 0.019 |
|  |  | HLA-DPA1 | -0.956 | 0.044 |
|  |  | LIME1 | 0.955 | 0.045 |
|  | Gamma delta T cell | CD36 | 0.998 | 0.002 |
|  |  | MAPK7 | 0.996 | 0.004 |
|  |  | MEIS3P1 | 0.965 | 0.035 |
|  |  | FABP1 | 0.952 | 0.048 |
|  |  | RPS24 | -0.966 | 0.035 |
|  |  | CCL13 | 0.983 | 0.017 |
|  | Immature B cell | CD22 | 0.968 | 0.032 |
|  |  | CYBB | -0.986 | 0.014 |
|  |  | ZCCHC2 | -0.972 | 0.028 |
|  | Memory B cell | RUNX2 | 0.954 | 0.046 |
|  | Regulatory T cell | CD72 | 0.982 | 0.018 |
|  |  | L1CAM | -0.993 | 0.007 |
|  |  | LIPA | 0.965 | 0.035 |
|  |  | MNDA | 0.965 | 0.035 |
|  |  | PELO | 0.984 | 0.016 |
|  |  | STAB1 | 0.956 | 0.044 |
|  | T follicular helper cell | B3GAT1 | 0.978 | 0.022 |
|  |  | CTSS | -0.979 | 0.021 |
|  |  | DPP4 | 0.979 | 0.021 |
|  |  | LRRC32 | 0.986 | 0.014 |
|  |  | NRP1 | 0.980 | 0.020 |
|  |  | RAE1 | 0.955 | 0.045 |
|  |  | LGMN | 1.000 | 0.000 |
|  | Type 1 T helper cell | BST1 | 0.960 | 0.041 |
|  |  | CD47 | -0.951 | 0.049 |
|  |  | DAB1 | 0.971 | 0.030 |
|  |  | THUMPD2 | 0.965 | 0.036 |
|  |  | RGS16 | 0.980 | 0.020 |
|  |  | P2RX5 | 0.971 | 0.029 |
|  |  | IRF1 | -0.958 | 0.042 |
|  |  | CALD1 | 0.999 | 0.001 |
|  | Type 17 T helper cell | ABCB1 | 0.991 | 0.009 |
|  |  | CAMTA1 | 0.977 | 0.023 |
|  | Type 2 T helper cell | DAPK1 | 0.960 | 0.040 |
|  |  | DNAJC12 | 0.973 | 0.027 |
|  |  | GNAI1 | 0.960 | 0.040 |
|  |  | NRP2 | 0.968 | 0.032 |
|  |  | PHLDA1 | 0.997 | 0.003 |
|  |  | RAB27B | 0.984 | 0.016 |
|  |  | HELLS | 0.990 | 0.010 |
|  |  | IL26 | 0.970 | 0.030 |
| Innate | Activated dendritic cell | ATP5B | 0.969 | 0.031 |
|  |  | TREM1 | 0.980 | 0.020 |
|  |  | SLC25A37 | -0.995 | 0.005 |
|  | CD56bright natural killer cell | MLST8 | 0.953 | 0.047 |
|  |  | CSTB | 0.984 | 0.016 |
|  |  | HDC | 0.984 | 0.016 |
|  |  | HOXA1 | 0.974 | 0.026 |
|  |  | HS2ST1 | -0.955 | 0.045 |
|  |  | HS3ST1 | 0.965 | 0.035 |
|  |  | CDH3 | 0.987 | 0.013 |
|  | CD56dim natural killer cell | NOTCH3 | 0.982 | 0.018 |
