# Supplementary material for: Potential links between COVID-19 and periodontitis: a bioinformatic analysis based on GEO datasets
Source: BMC Oral Health. 2022 Nov 21;22:520. doi: 10.1186/s12903-022-02435-4 (PMC9682728; doi:10.1186/s12903-022-02435-4)

**Supplement 5**

Bioinformatic procedure of this study was listed step-by-step

① The data source

As we described in the **Materials & methods** part, “COVID-19 (GSE164805) and periodontitis (GSE12484) were obtained from the GEO database in NCBI (<https://www.ncbi.nlm.nih.gov/geo/>)” We can get the detailed information about the dataset, with the blue button of GEO2R, the GEO Online Analysis Tool, at the bottom of the page, as follows.

**COVID-19 (GSE164805)**


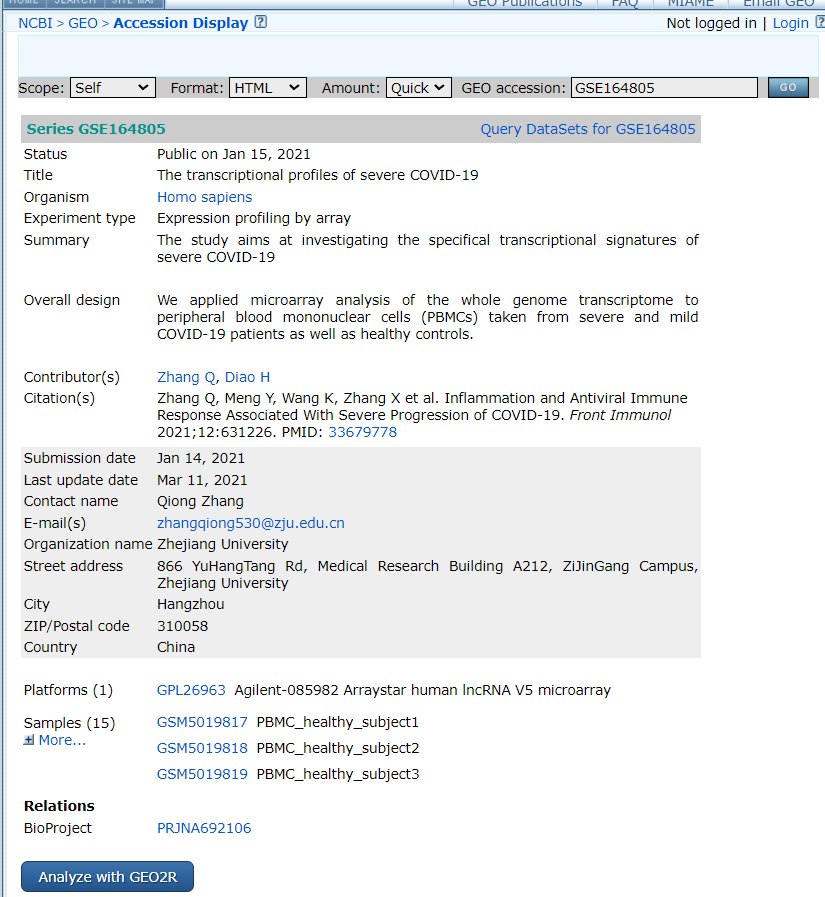


**periodontitis (GSE12484)**


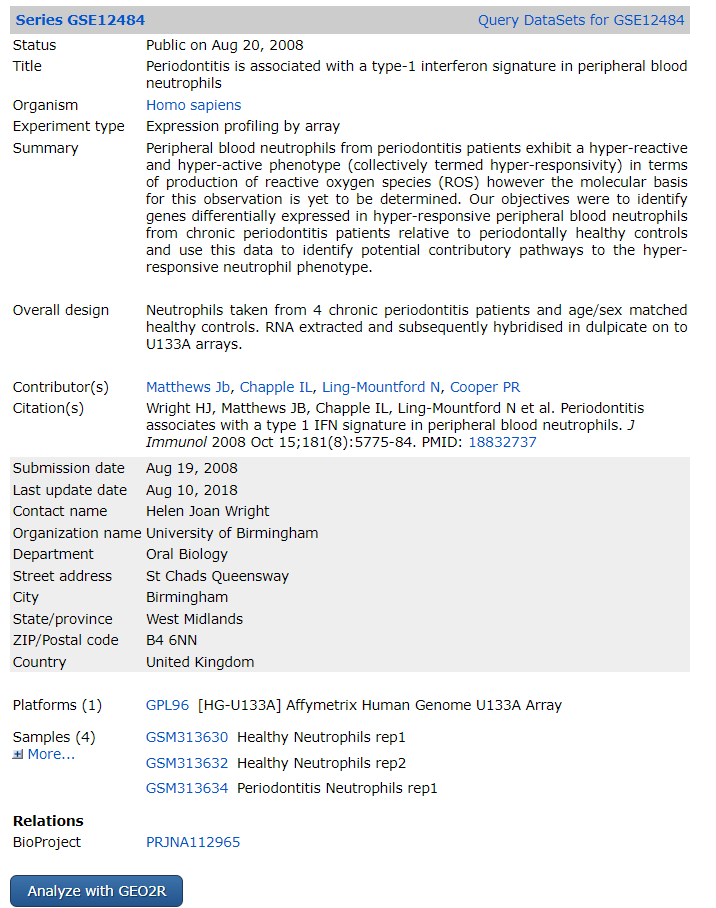


②**Differentially expressed genes analysis**

Then to obtain the differentially expressed genes, “GEO2R, the GEO Online Analysis Tool (<http://www.ncbi.nlm.nih.gov/geo/geo2r/>), was used to identify DEGs in GSE164805 and GSE12484 between the diseased groups and control groups.”

For example: Click the blue button “Analyze with GEO2R”. After entering the page, the data set was divided into “COVID-19 group” and “normal group” in according to title in GSE164805, and “periodontitis group” and “normal group” according to title in GSE12484, then click the bule button “Analyze” at the bottom of the page.

**COVID-19 (GSE164805)**


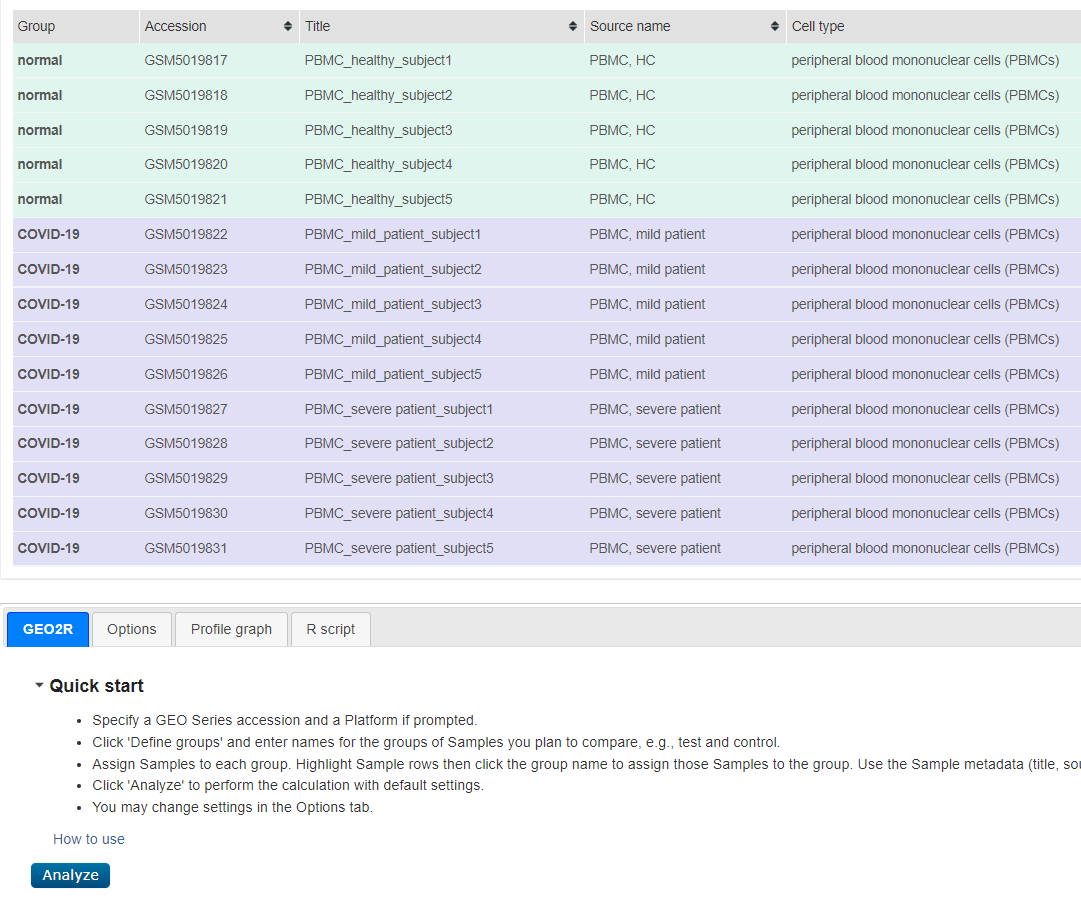


**periodontitis (GSE12484)**


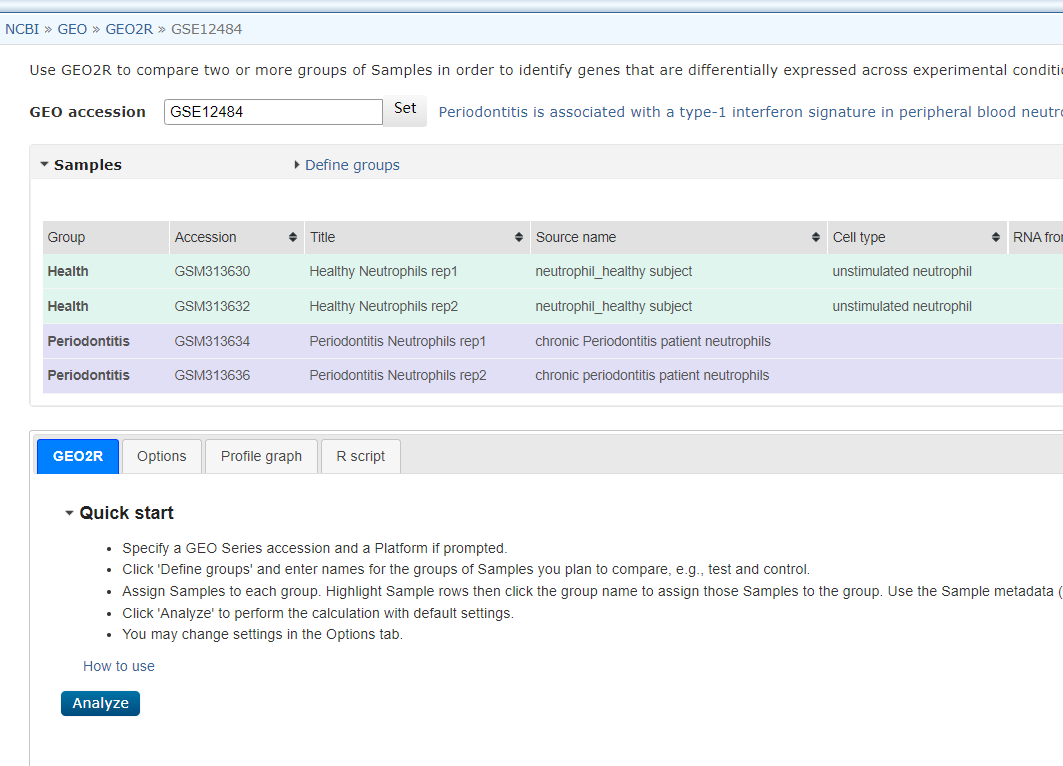


We choose GSE164805 to make further demonstrations. Next, click the bottom “Download full table” to get the gene expression profile


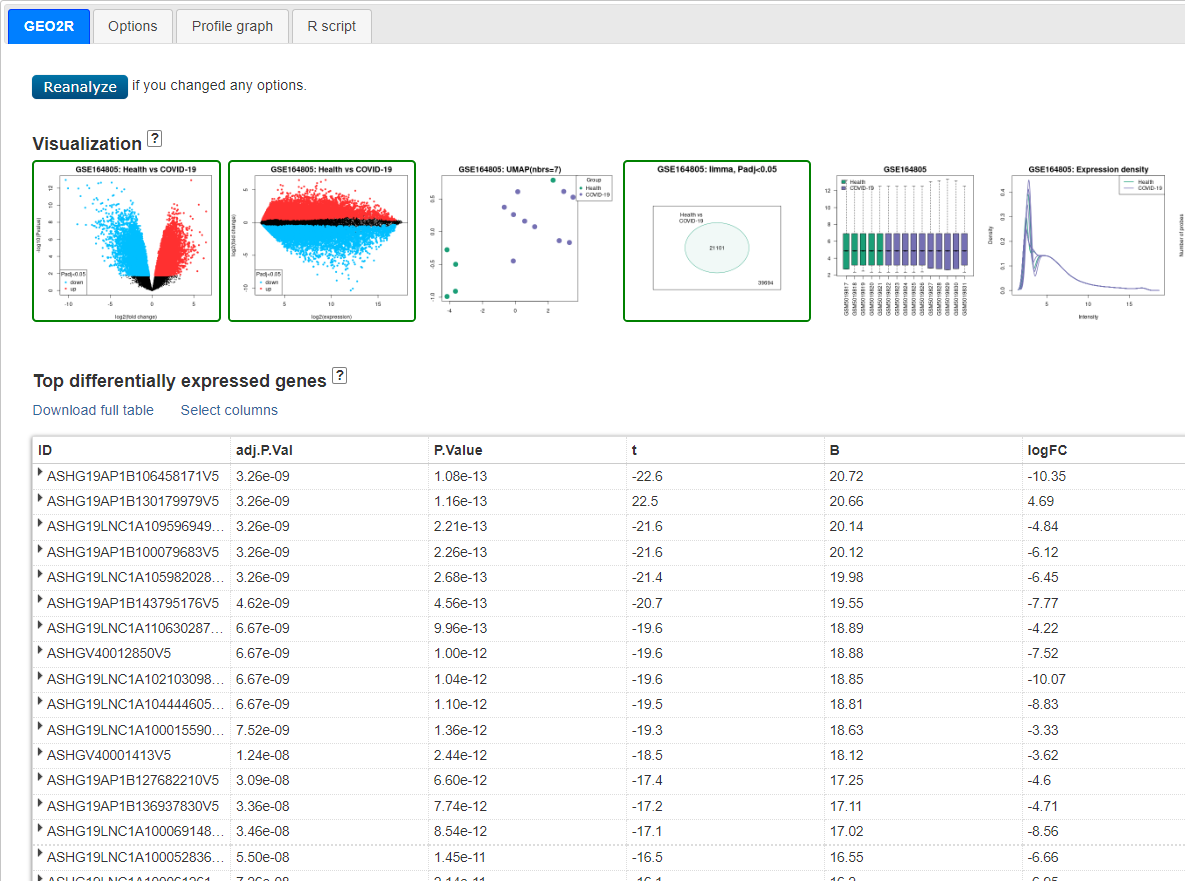


The differentially expressed gene expression profile (**Table a**):


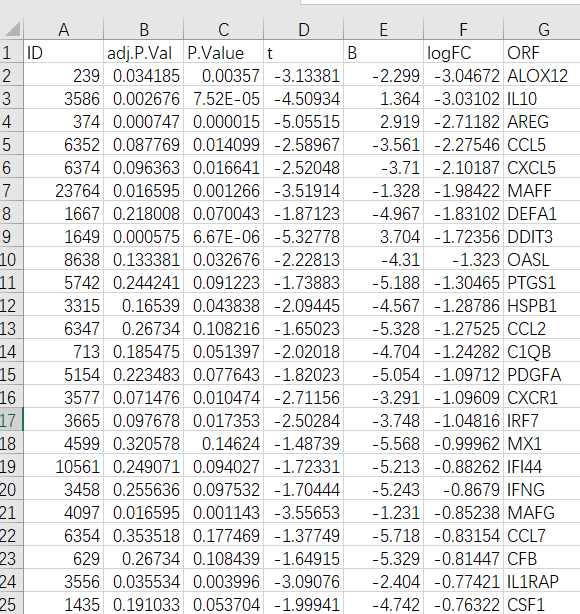


According to the evaluation criteria of adjust P-value <0.05 and |log2(FC)|>2.0, we can get the DEGs.


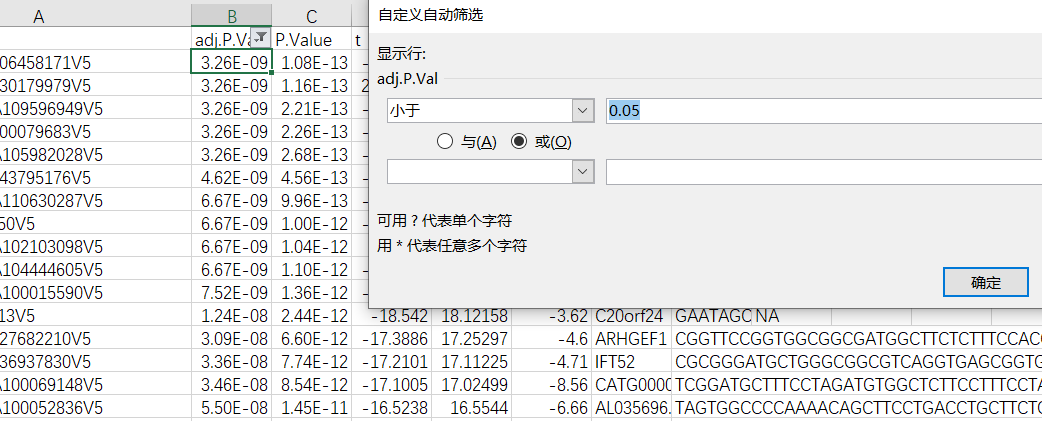


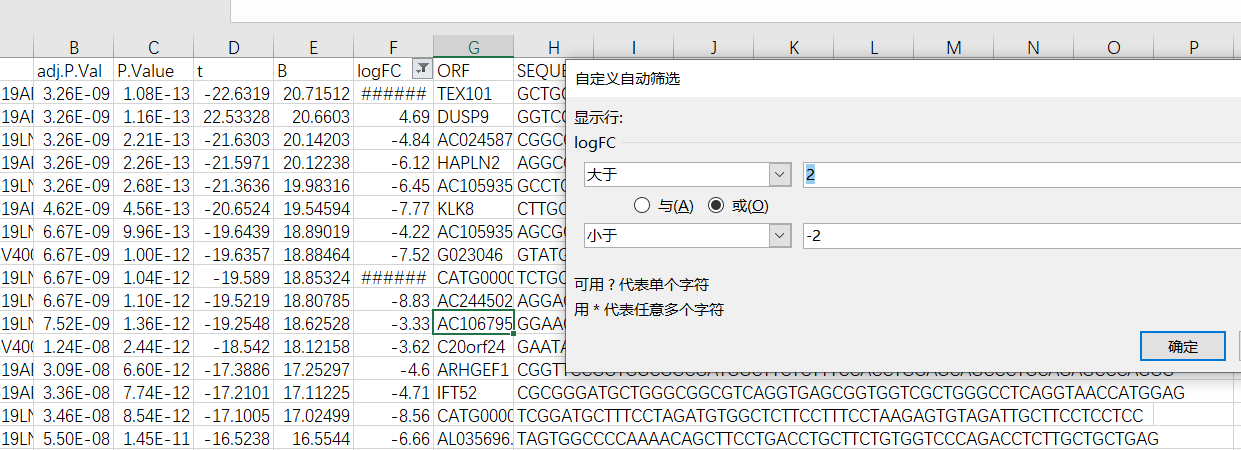


**To map the volcanoplot,** we need to extract 2 columns of data (adjust P-value and log2(FC)) from the above **Table a.** As follows, the table is in txt format.


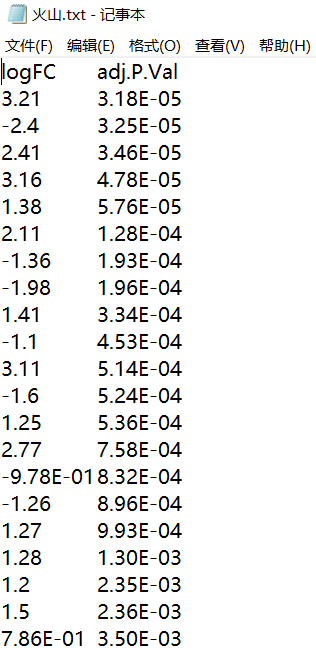


As we stated in the acknowledgement, “a free online platform (http://www.bioinformatics.com.cn) for data analysis and visualization.” Next, Login to this website (<http://www.bioinformatics.com.cn/>) to upload the data. We can get the volcanoplot.


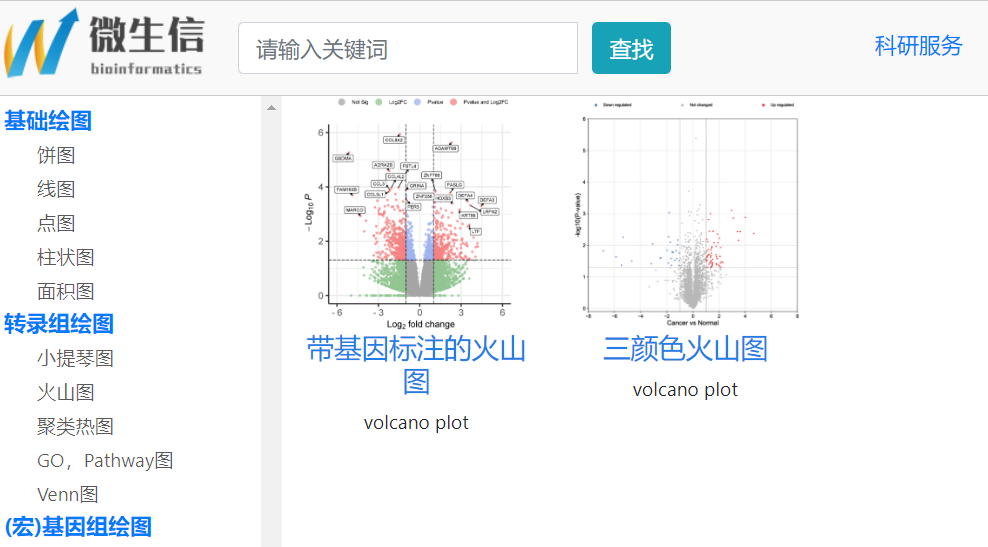


**To map the** **heatmap**, we need to extract the gene expression profile including the 20 genes with the highest up- or down-regulation of expression each. As follows, the table is in txt format.


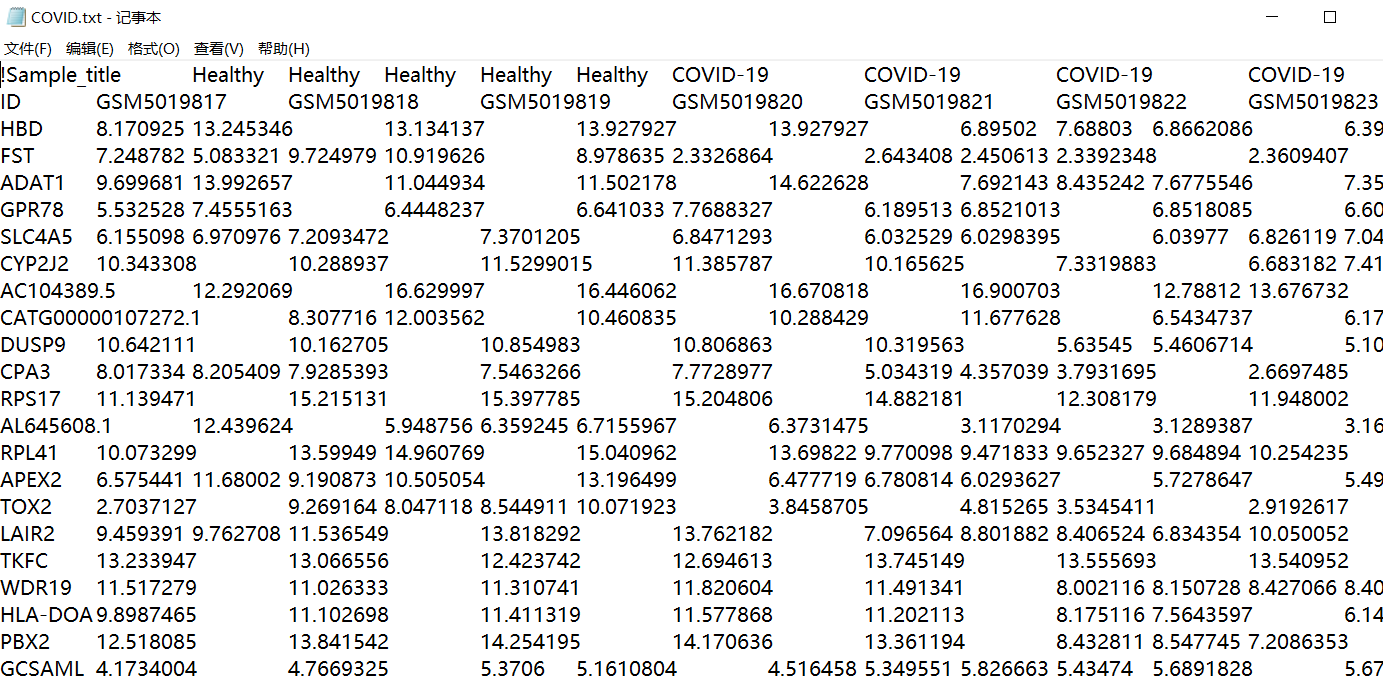


Next, Login to this website (<http://www.bioinformatics.com.cn/>) to upload the data. We can get the heatmap.


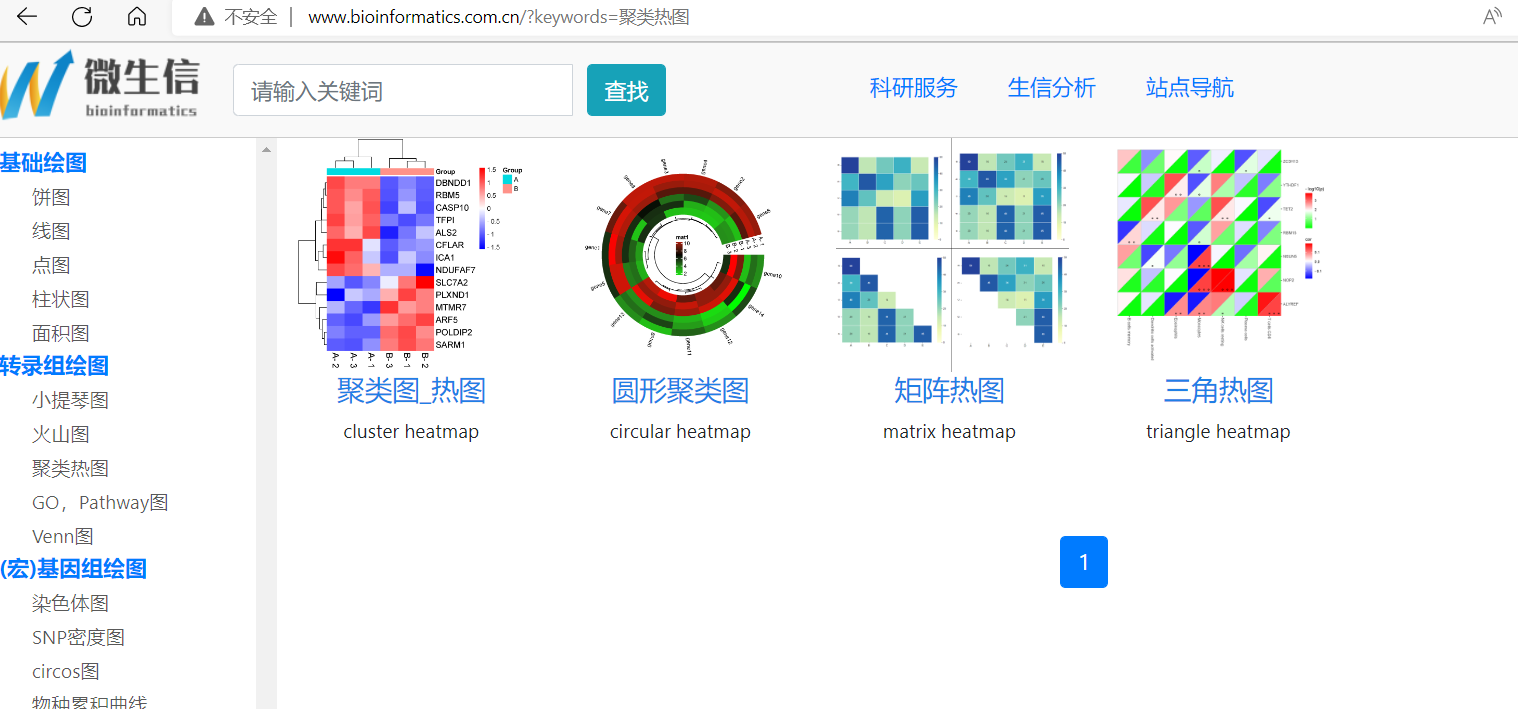


③ “BioGPS database (<http://biogps.org/>) was screened to find the tissue-specific expression of the intersected DEGs.”

Login to this website (<http://biogps.org/>) to enter the intersected DEGs, click the “Search” bottom.


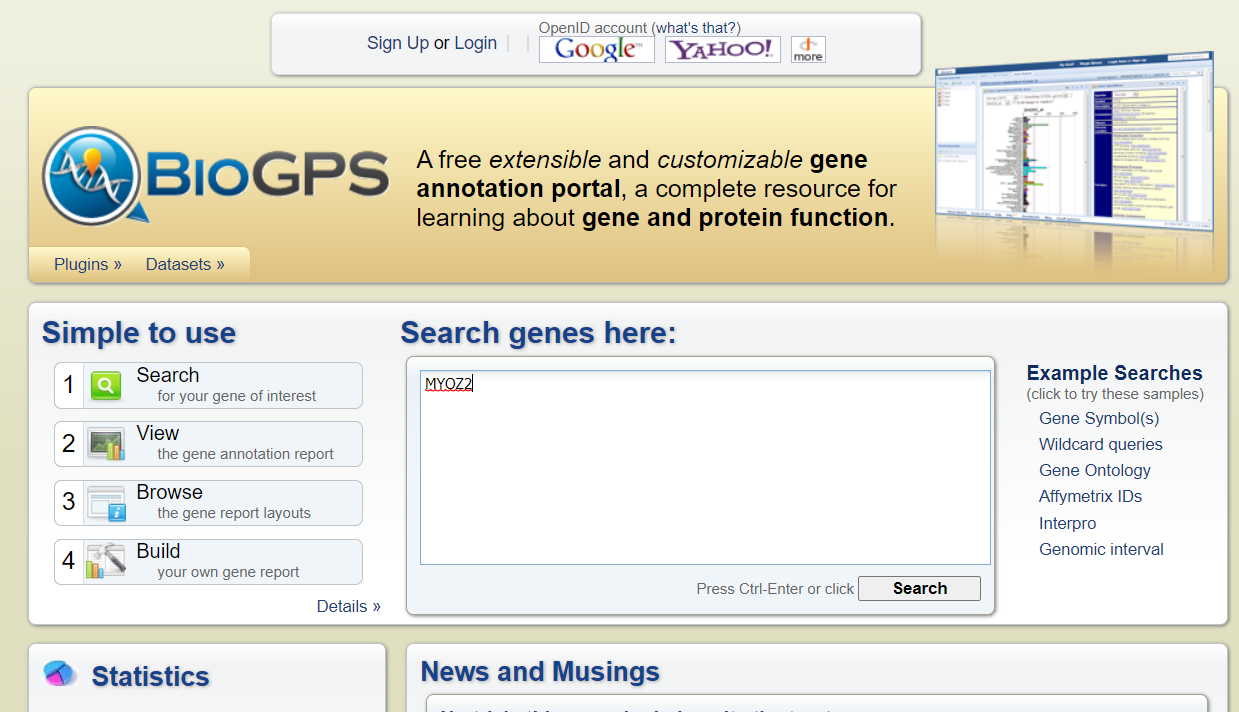


Then we choose the human species.


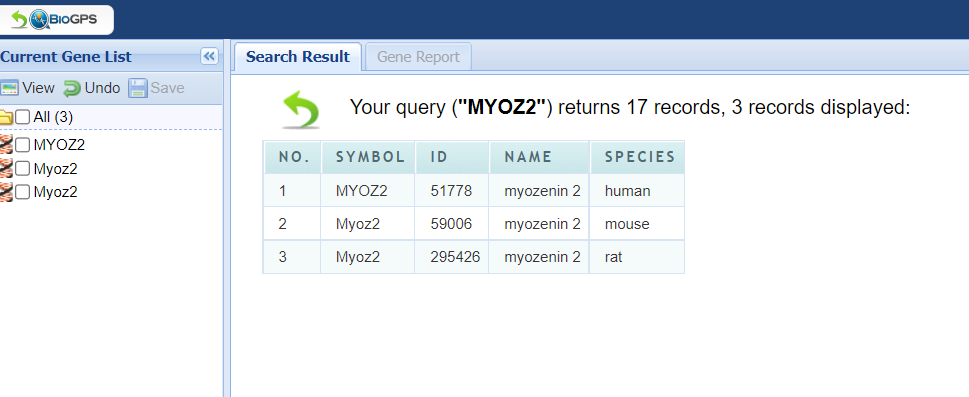


We can obtain the expression of MYOZ2 in different tissue, and we select the tissue with the highest MYOZ2 expression.


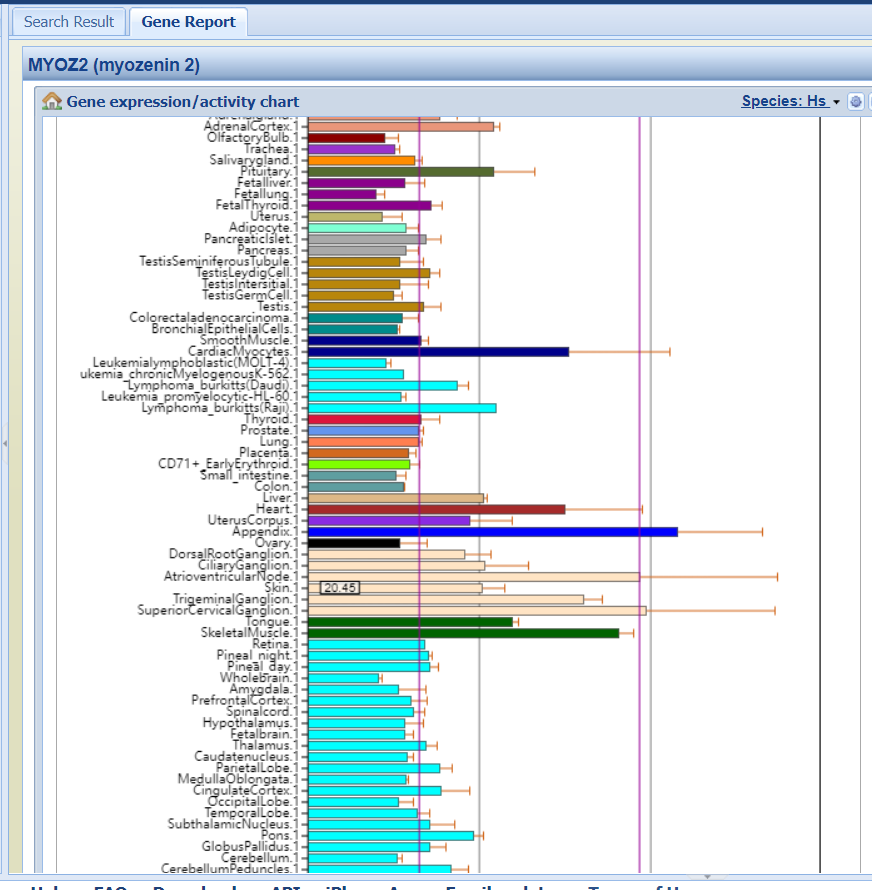


④ **Functional enrichment analysis of DEGs**

As we described, “Gene Ontology (GO) analysis and Kyoto Encyclopedia of Genesand Genomes (KEGG) pathways enrichment were conducted through Metascape database (<https://metascape.org/>).”

Login to this website (<https://metascape.org/>) to enter the 56 intersected DEGs, click the “Expression Analysis” bottom.


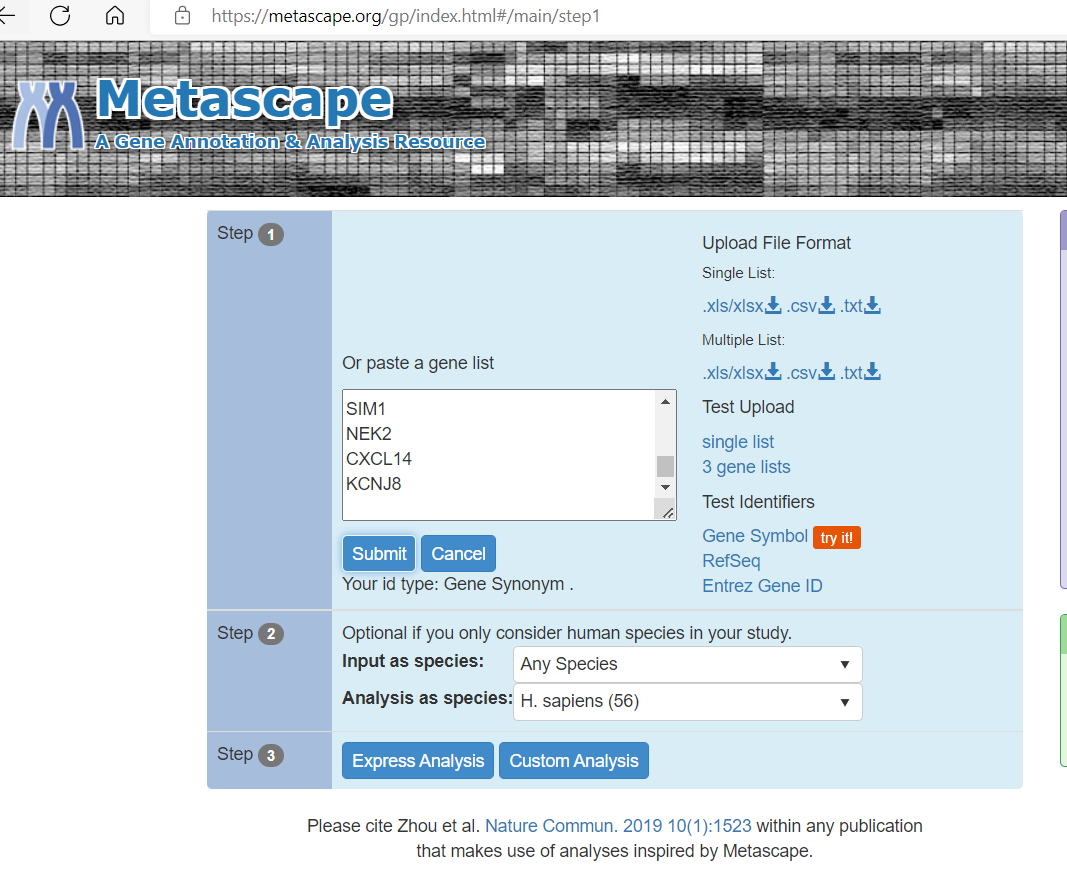


Then, click the “Gene List Report Excel Sheets” to download the results in excel format.


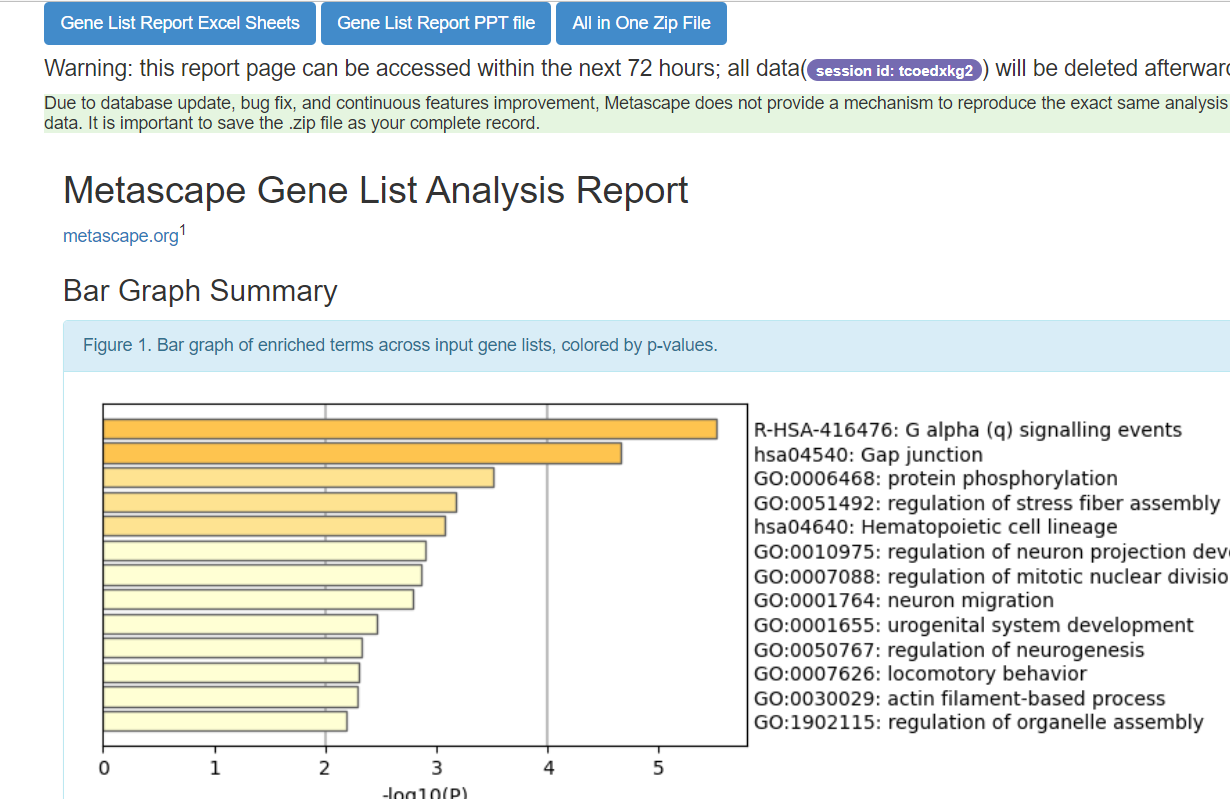


We can obtain the results of GO/KEGG analysis. Then, we delete the repetitions and select the 10 GO terms with the lowest *P* value.


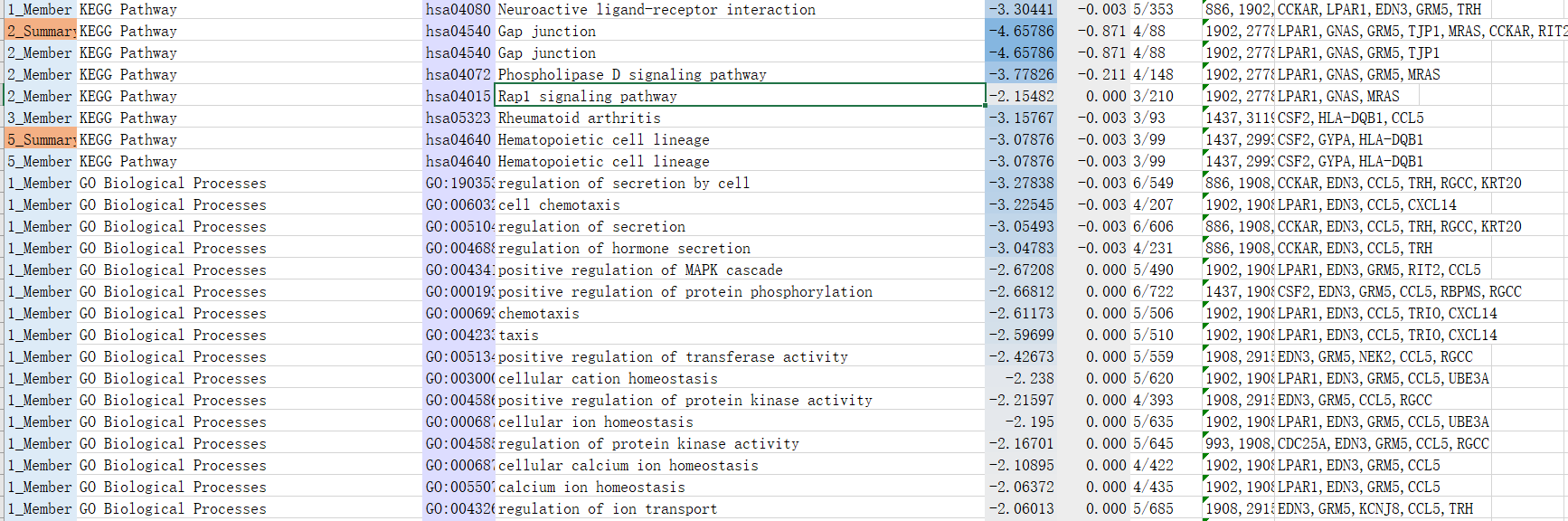


After that Login to this website (<http://www.bioinformatics.com.cn/>) to upload the data. Finally, we get the GO/KEGG figures.


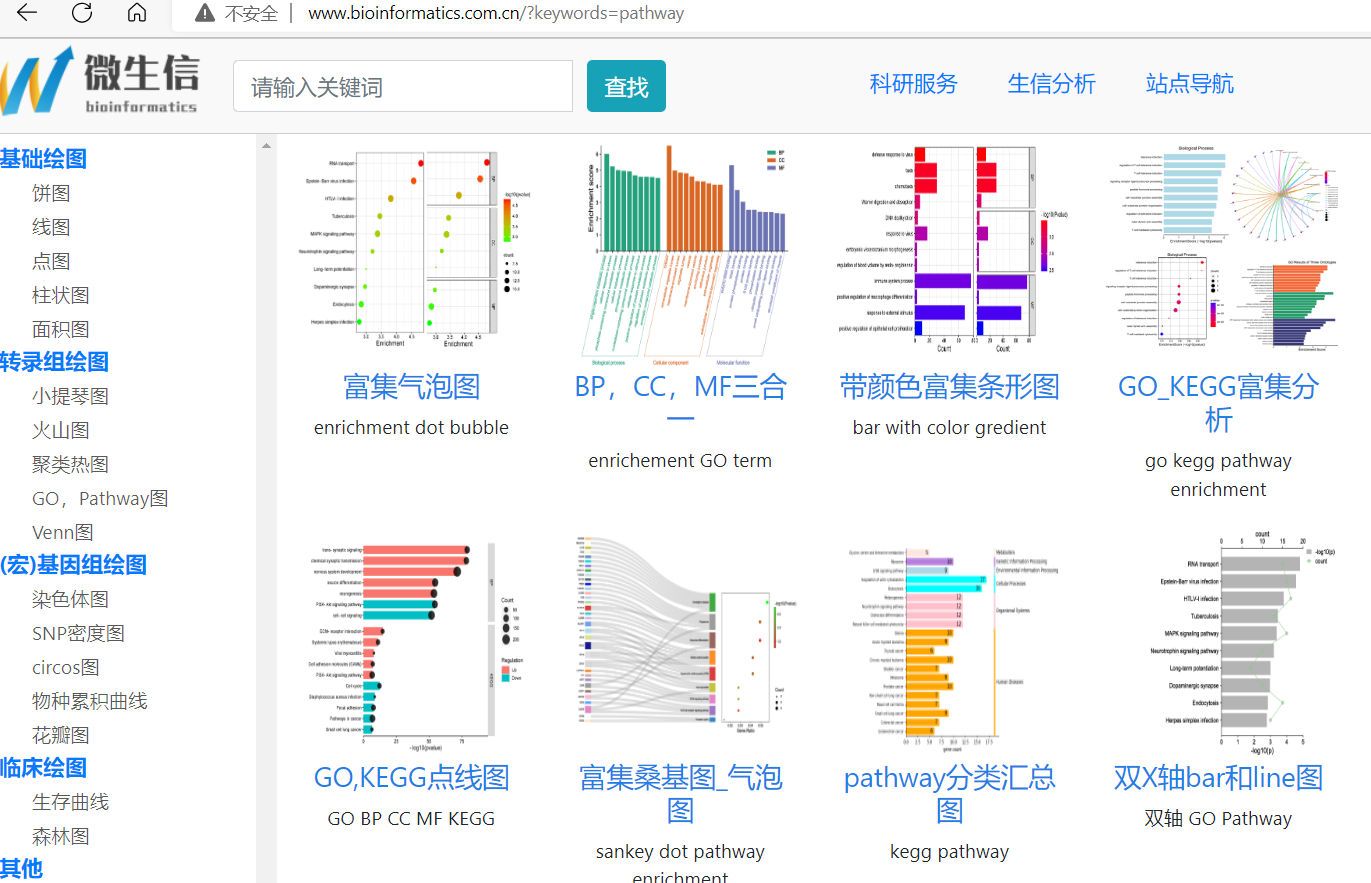


⑤PPInetwork

As we described, “STRING database (https://string-db.org/) was applied to identify protein-protein interactions (PPI). Cytoscape was used to visualize the PPI. Furthermore, CytoHubba, a plug-in of Cytoscape, was applied to find the top 5 hub genes through Maximal Clique Centrality algorithm.”

Login to this website (https://string-db.org/) to enter the 56 intersected DEGs, click the “Search” bottom.


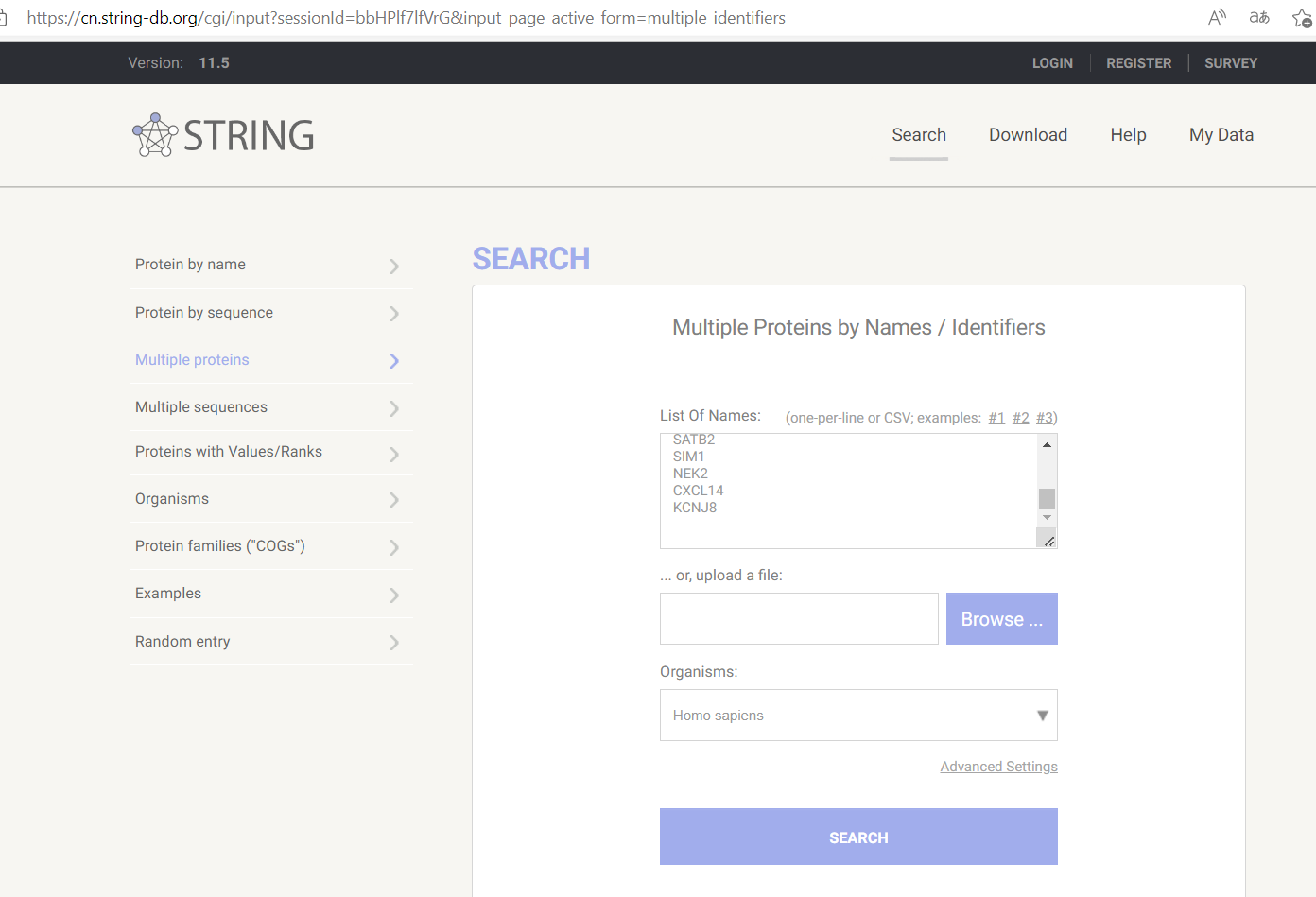


Then, click the “Download” to download the results in excel format.


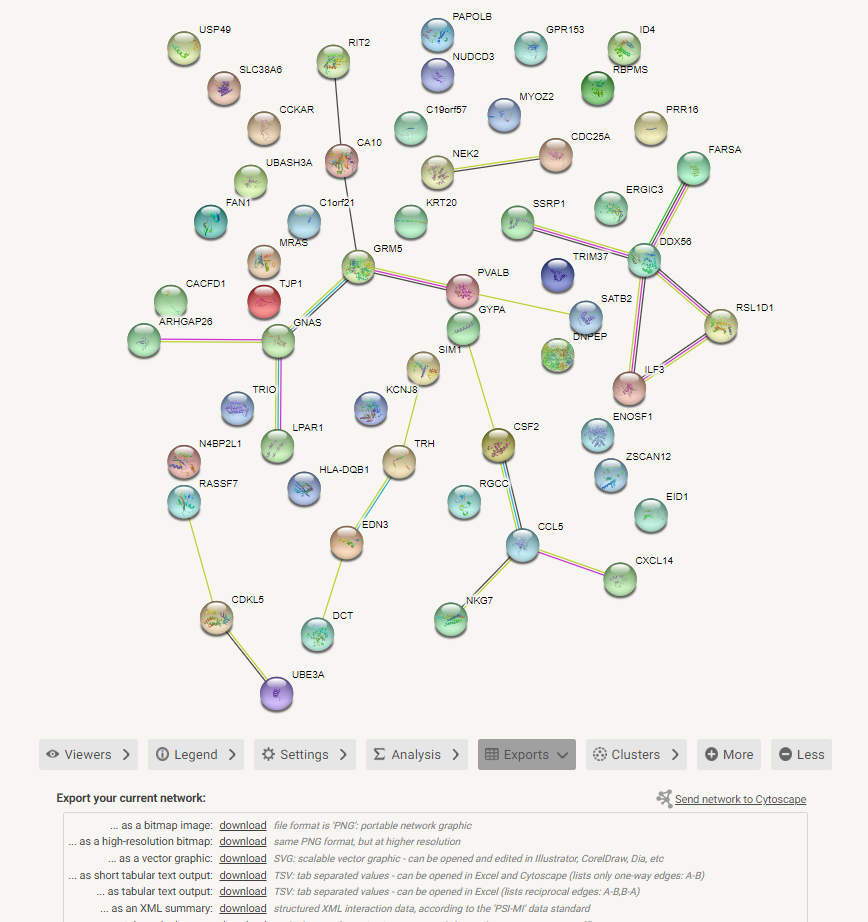


Cytoscape was used to visualize the PPI.


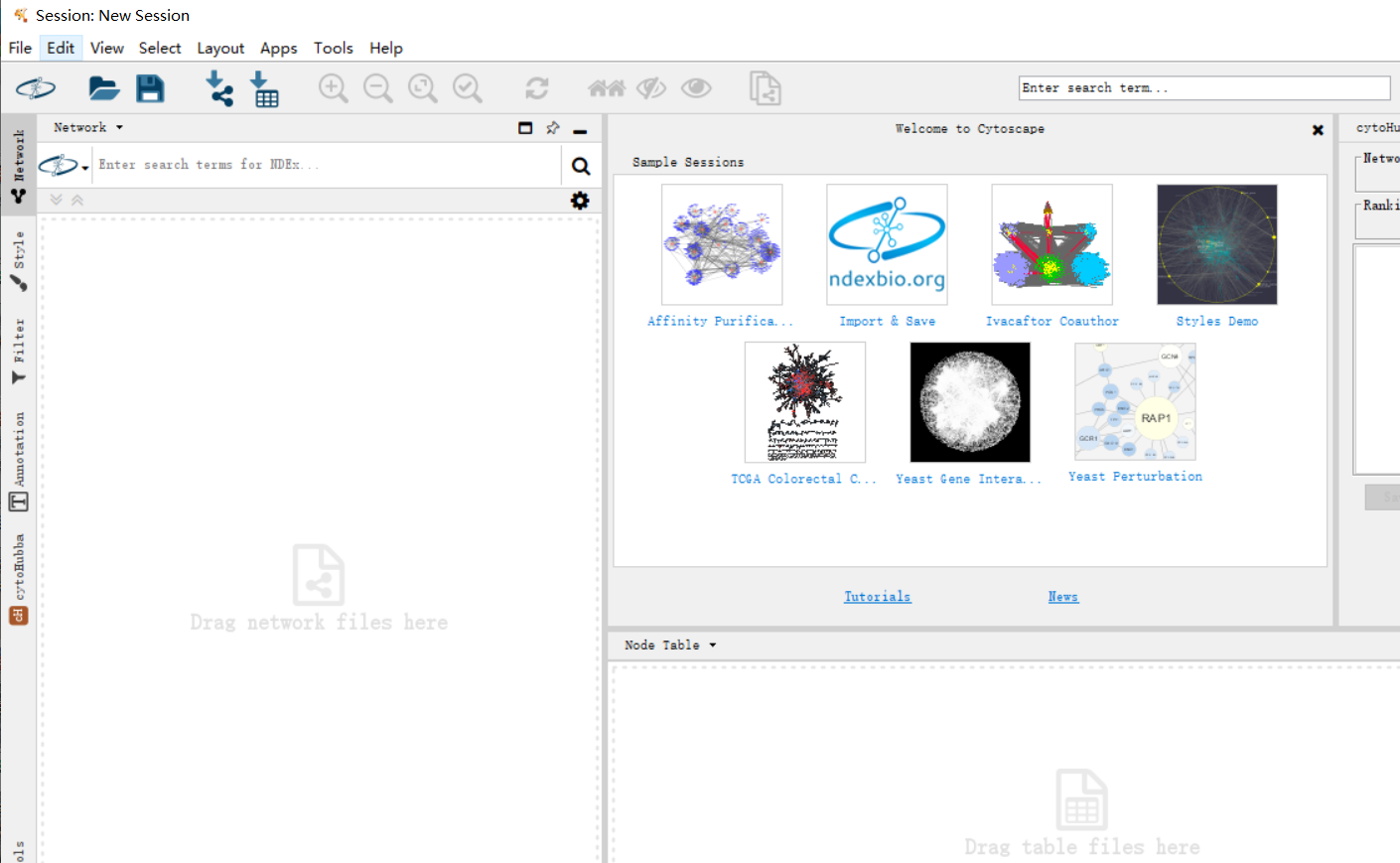


CytoHubba, a plug-in of Cytoscape, was applied to find the top 5 hub genes through Maximal Clique Centrality algorithm.


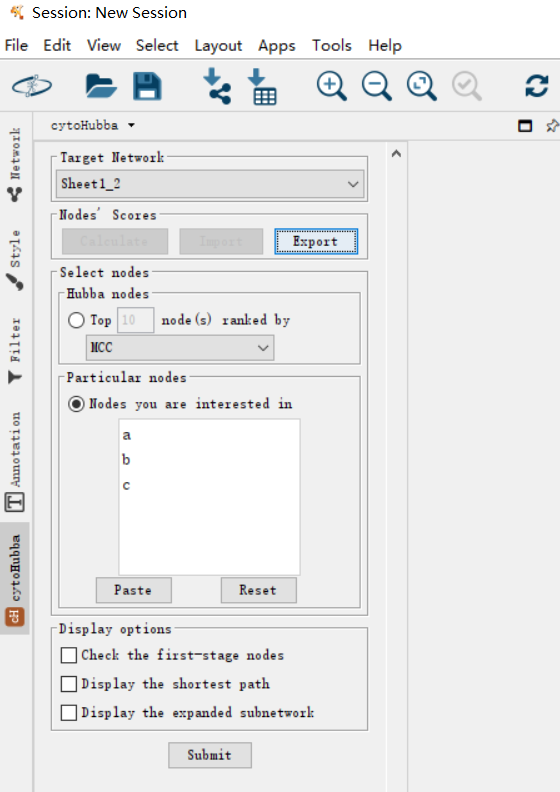


**⑥Interacted mRNA-miRNA network**

The miRDB database (http://mirdb.org/),[20] miRTarbase database (https://mirtarbase.cuhk.edu.cn/)[21] and Targetscan database (https://www.targetscan.org/)[22] were screened to identify the miRNAs interacted with the top 5 hub genes. The miRNA-mRNA network was built through the Cytoscape software.

miRDB database


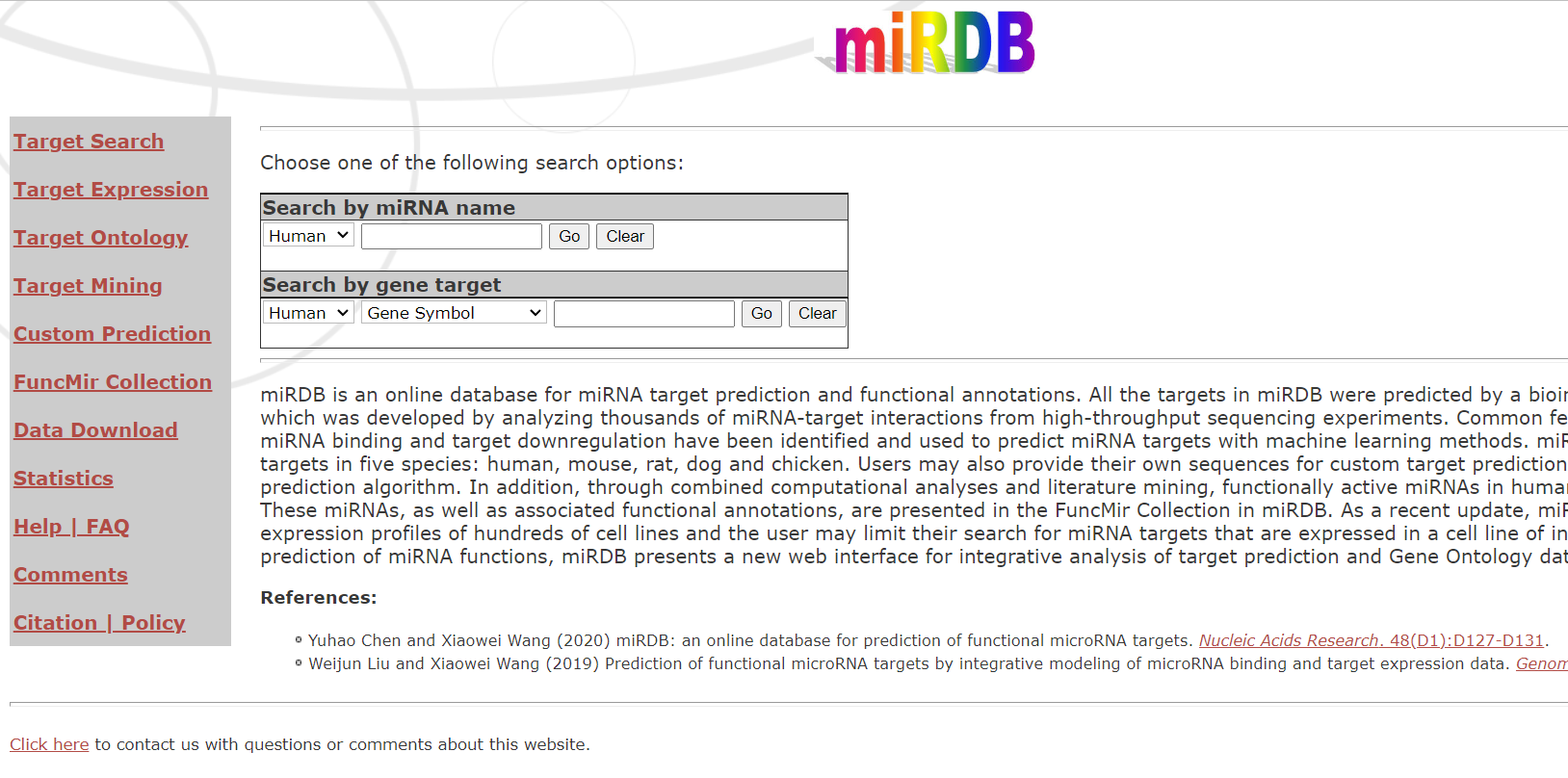


miRTarbase database


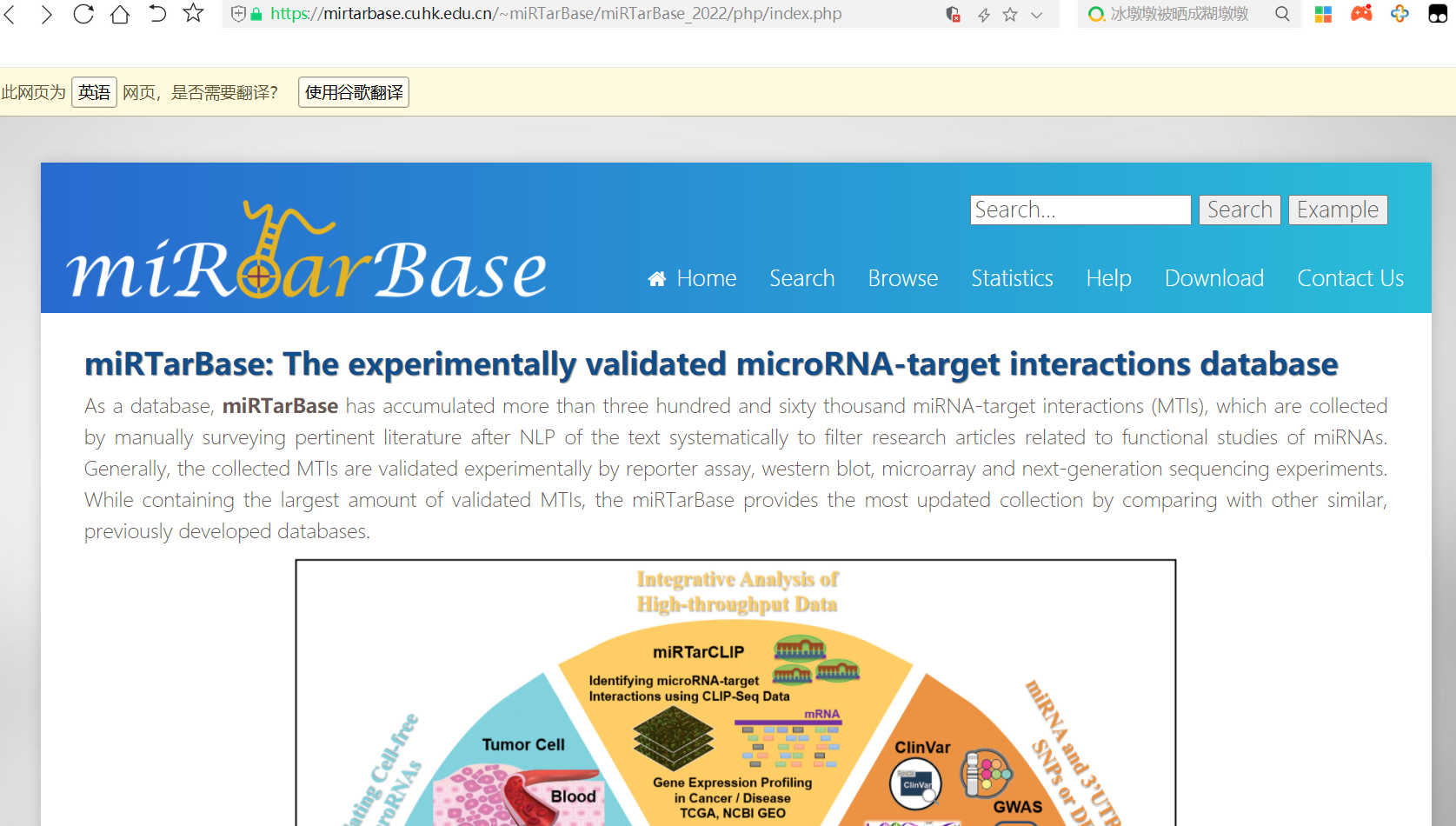


Targetscan database


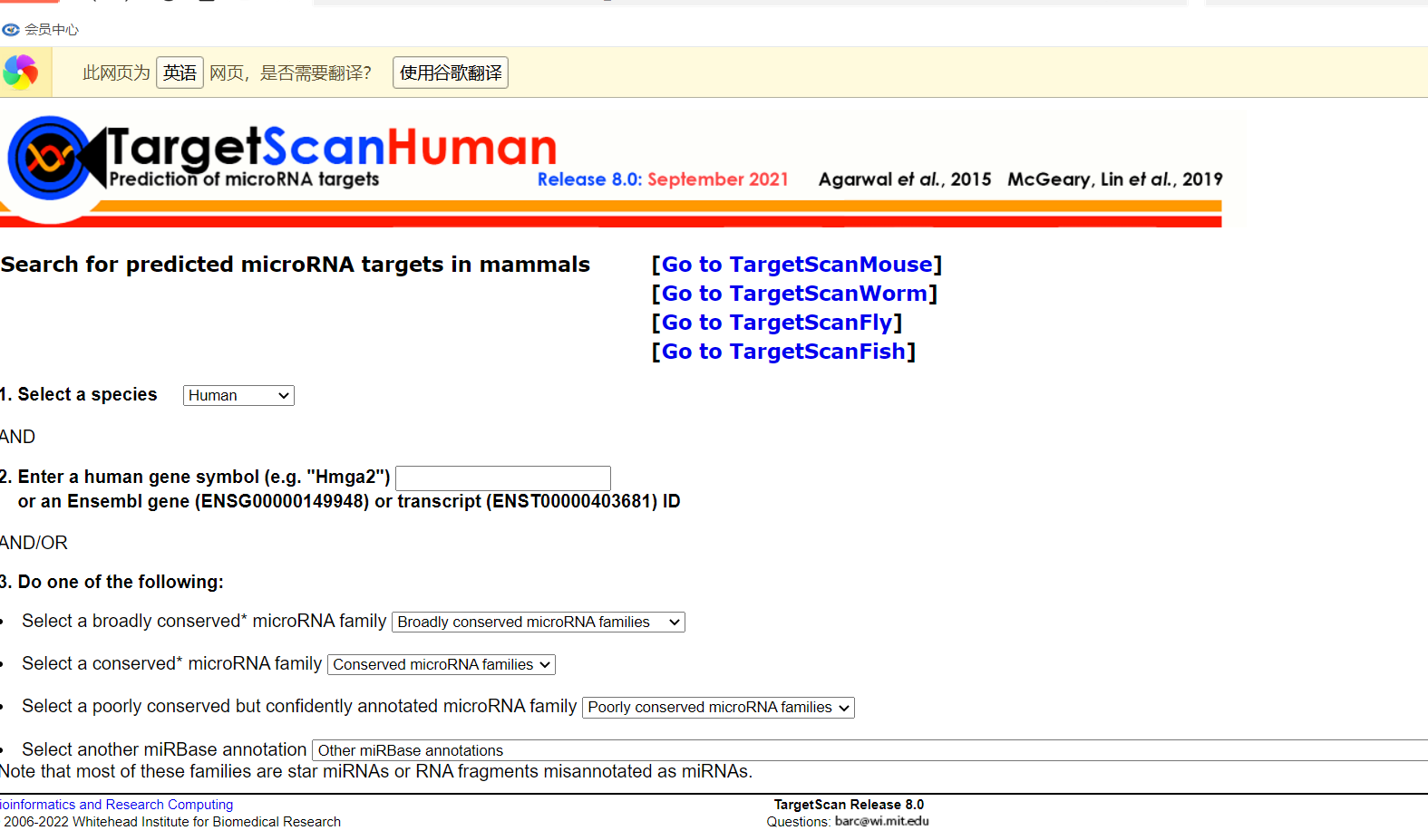

Supplement: Supplementary file 5 — Additional file 5: Bioinformatic procedure. [file 12903_2022_2435_MOESM5_ESM.docx]
